# Regulating off-centering distortion maximizes photoluminescence in halide perovskites

**DOI:** 10.1093/nsr/nwaa288

**Published:** 2020-11-30

**Authors:** Xujie Lü, Constantinos Stoumpos, Qingyang Hu, Xuedan Ma, Dongzhou Zhang, Songhao Guo, Justin Hoffman, Kejun Bu, Xiaofeng Guo, Yingqi Wang, Cheng Ji, Haijie Chen, Hongwu Xu, Quanxi Jia, Wenge Yang, Mercouri G Kanatzidis, Ho-Kwang Mao

**Affiliations:** Centerfor High Pressure Science and Technology Advanced Research (HPSTAR), Shanghai 201203, China; Department of Chemistry, Northwestern University, Evanston, IL 60208, USA; Department of Materials Science and Technology, Voutes Campus, University of Crete, Heraklion GR-70013, Greece; Centerfor High Pressure Science and Technology Advanced Research (HPSTAR), Shanghai 201203, China; Center for Nanoscale Materials, Argonne National Laboratory, Lemont, IL 60439, USA; Partnership for Extreme Crystallography, University of Hawaii at Manoa, Honolulu, HI 96822, USA; Centerfor High Pressure Science and Technology Advanced Research (HPSTAR), Shanghai 201203, China; Department of Chemistry, Northwestern University, Evanston, IL 60208, USA; Centerfor High Pressure Science and Technology Advanced Research (HPSTAR), Shanghai 201203, China; Department of Chemistry and Alexandra Navrotsky Institute for Experimental Thermodynamics, Washington State University, Pullman, WA 99164, USA; Centerfor High Pressure Science and Technology Advanced Research (HPSTAR), Shanghai 201203, China; Centerfor High Pressure Science and Technology Advanced Research (HPSTAR), Shanghai 201203, China; Department of Chemistry, Northwestern University, Evanston, IL 60208, USA; Earth and Environmental Sciences Division, Los Alamos National Laboratory, Los Alamos, NM 87545, USA; Department of Materials Design and Innovation, University at Buffalo—The State University of New York, Buffalo, NY 14260, USA; Centerfor High Pressure Science and Technology Advanced Research (HPSTAR), Shanghai 201203, China; Department of Chemistry, Northwestern University, Evanston, IL 60208, USA; Centerfor High Pressure Science and Technology Advanced Research (HPSTAR), Shanghai 201203, China

**Keywords:** halide perovskites, high pressure, off-centering distortion, optical properties, lone-pair electrons, quantitative relationship

## Abstract

Metal halide perovskites possess unique atomic and electronic configurations that endow them with high defect tolerance and enable high-performance photovoltaics and optoelectronics. Perovskite light-emitting diodes have achieved an external quantum efficiency of over 20%. Despite tremendous progress, fundamental questions remain, such as how structural distortion affects the optical properties. Addressing their relationships is considerably challenging due to the scarcity of effective diagnostic tools during structural and property tuning as well as the limited tunability achievable by conventional methods. Here, using pressure and chemical methods to regulate the metal off-centering distortion, we demonstrate the giant tunability of photoluminescence (PL) in both the intensity (>20 times) and wavelength (>180 nm/GPa) in the highly distorted halide perovskites [CH_3_NH_3_GeI_3_, HC(NH_2_)_2_GeI_3_, and CsGeI_3_]. Using advanced *in situ* high-pressure probes and first-principles calculations, we quantitatively reveal a universal relationship whereby regulating the level of off-centering distortion towards 0.2 leads to the best PL performance in the halide perovskites. By applying this principle, intense PL can still be induced by substituting CH_3_NH_3_^+^ with Cs^+^ to control the distortion in (CH_3_NH_3_)_1-x_Cs_x_GeI_3_, where the chemical substitution plays a similar role as external pressure. The compression of a fully substituted sample of CsGeI_3_ further tunes the distortion to the optimal value at 0.7 GPa, which maximizes the emission with a 10-fold enhancement. This work not only demonstrates a quantitative relationship between structural distortion and PL property of the halide perovskites but also illustrates the use of knowledge gained from high-pressure research to achieve the desired properties by ambient methods.

## Teaser

By regulating the highly distorted halide perovskites using pressure, a quantitative relationship between structural distortion and emission property is demonstrated. The extracted principle is applied to materials design and the results give further support to the revealed relationship.

## INTRODUCTION

Halide perovskites have exhibited extraordinary electronic and optical properties including high absorption coefficients, long carrier lifetimes and large charge diffusion lengths, that lead to a range of applications in low-cost and high-efficiency photovoltaic devices, light-emitting diodes, lasers and photodetectors [1–5]. Bright photoluminescence (PL) has been achieved in lead halide perovskites and further enhancements have been realized by compositional, dimensional and structural modifications, as well as post-treatments such as light exposure and surface passivation [2–9]. Despite the tremendous progress in exploring and optimizing halide perovskites in the past several years, many fundamental challenges need to be addressed in order to further refine the design principles for excellent properties and thus fully utilize their unique functionalities for future technological applications. For example, the intensity of light emission has been reported to be related to the structural distortion of perovskite structures [5,10,11], yet without a systematic investigation of the structure-property relationships. Achieving a deeper understanding requires suitable material systems in combination with advanced *in situ*/*operando* characterization tools.

**Figure 1.**
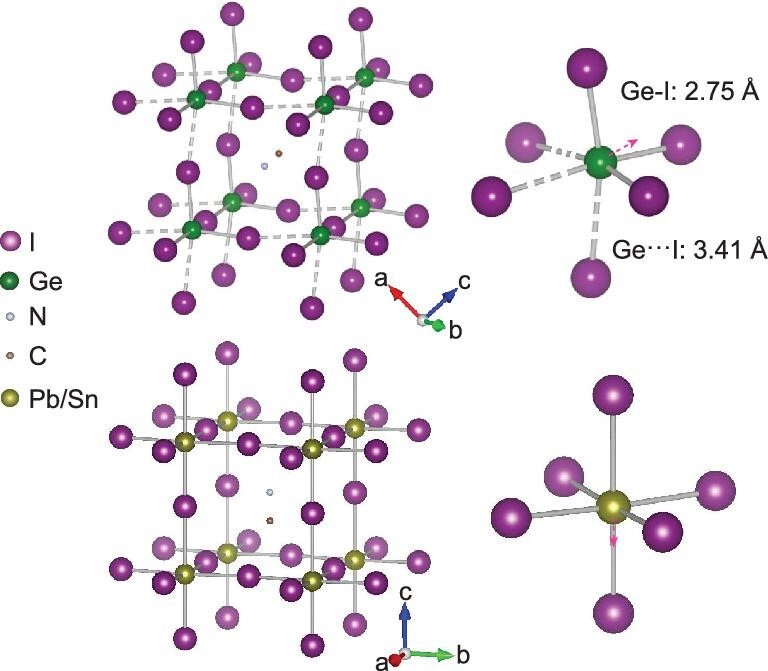
Crystal structure of MAGeI_3_ in comparison with the Pb/Sn analog. MAGeI_3_ crystallizes in a rhombohedral structure with a large distortion of GeI_6_ octahedra which is due to the displacement of Ge along all the three directions of I-Ge-I bonds in the octahedron (i.e. along the normal direction of octahedral face). Such distortion creates two sets of short and long Ge-I bonds with a length difference as high as 24%. In comparison, both MAPbI_3_ and MASnI_3_ perovskites have a tetragonal structure, where the structural distortion is minor.

Unlike the Pb and Sn congeners, germanium halide perovskite (CH_3_NH_3_GeI_3_) crystallizes in a polar space group *R*3*m*. The large size difference and electronegativity mismatch between Ge^2+^ and I^−^ gives rise to high polarizability and large structural distortion [12,13]. The structure of this perovskite, shown in Fig. 1, has a highly distorted GeI_6_ octahedron, where Ge stays away from the proper center and forms three short Ge-I bonds and three long Ge···I bonds. This metal off-centering is a consequence of the strong stereochemical activity of the 4*s*^2^ lone-pair electrons in Ge^2+^. The two sets of Ge-I bond distances (2.75 and 3.41 Å) exhibit a large difference of 24%. In comparison, the differences in the (Pb/Sn)-I bond lengths in the Pb and Sn compounds are 3% and 5%, respectively [6]. Consequently, the highly distorted structure of CH_3_NH_3_GeI_3_ (hereafter MAGeI_3_) leads to unusual characteristics and unique optical properties. For instance, MAGeI_3_ has an anomalously wider band gap of 1.9 eV, relative to 1.3 eV for MASnI_3_ and 1.6 eV for MAPbI_3_ [12–14]. Moreover, MAGeI_3_ shows no detectable PL at ambient conditions while both the Sn and Pb compounds exhibit strong PL. The large degree of distortion in the Ge perovskite, setting it apart from the Sn and Pb analogs, provides tremendous new opportunities for the fundamental understanding of the interplay between the structural distortion and properties.

The principal mechanism generating the multi-functional nature of halide perovskites is the competitive interplay between the electron, orbital and atomic lattice degrees of freedom across similar energy scales [15–18]. These degrees of freedom can be effectively tuned by applying external stimuli, including temperature, pressure, and electric and magnetic fields. Pressure, as a thermodynamic parameter, can effectively modify the lattice and electronic configurations of materials without changing their chemical compositions [19–22]. Pressure processing has not only been used to further our fundamental understanding and discover new physics, but also enabled the exploration of novel materials such as metastable nanophases [23–28]. The pressure effects should be more dramatic in the halide perovskites because of their dynamically flexible and soft lattices [29–34]. Here, we report the emergence of strong PL with giant tunability of the emission wavelength and intensity in germanium halide perovskites under compression. By employing *in situ* high pressure and synchrotron techniques along with first-principles calculations, we reveal the relationship between the structural distortion and PL property of these materials, and elucidate the underlying mechanisms of the dramatic pressure-induced changes. Using the gained knowledge, we successfully obtained mixed-cation perovskites with emergent high-pressure properties by normal synthetic methods, that is, using chemical tailoring to simulate the effects of external pressures.

## RESULTS AND DISCUSSION

**Figure 2.**
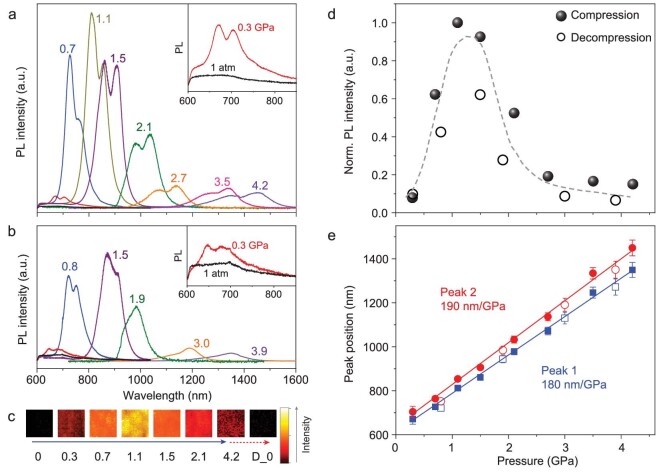
Pressure-dependent photoluminescence properties of MAGeI_3_. *In situ* PL spectra during (a) the compression and (b) decompression cycles. The insets show the zoomed-in spectra at 1 atm and 0.3 GPa. (c) Two-dimensional mappings of the PL signal at eight selected pressures, where the brighter color indicates the higher emission intensity. (d) The pressure dependence of spectrally integrated PL intensity. The highest emission is achieved at around 1 GPa. (e) PL peak position as a function of pressure, showing giant tunability of over 180 nm/GPa. For comparison, the tunability of the emission wavelength for the Pb analog is less than 40 nm/GPa.

Rhombohedral MAGeI_3_, which does not have a detectable emission at ambient conditions [12], exhibits an emergent and tunable PL by applying external pressure. Figure 2a and b shows the *in situ* PL spectra under various pressures during compression and decompression, respectively. Figure 2c shows the two-dimensional mappings of the PL signal at eight selected pressures, where the brighter color indicates the higher emission intensity. A clear PL signal emerges at just 0.3 GPa. The intensity increases dramatically with pressure and reaches the highest value at 1.1 GPa and subsequently decreases with further pressurization. A volcano shape of the pressure-dependent PL intensity is observed during both compression and decompression (Fig. 2d), where an increase of 20 times in the intensity can be achieved when the pressure increases from 0.3 to 1.1 GPa.

Intriguingly, the PL peak position of MAGeI_3_ changes dramatically with pressure, from below 700 nm at 0.3 GPa to over 1400 nm at 4.2 GPa (Fig. 2a and e). By fitting the pressure-dependent peak positions, the emission wavelength can be tuned by more than 180 nm/GPa. Such a tunability is enormous compared to the reported values of other halide perovskites as well as other known medium band-gap semiconductors. For instance, the pressure dependence of PL in MAPbI_3_ is less than 40 nm/GPa (Fig. S1) [35]. The giant tunability of MAGeI_3_ is presumably due to the large degree of off-centering distortion in the GeI_6_ octahedron with three short and three long Ge-I bonds (Fig. 1). Such a highly sensitive pressure response together with the good linear relationship between the PL peak position and the pressure (Fig. 2e) raises the prospect of applying this material in precise pressure and stress detectors.

The unique lattice and electronic structures of the MAGeI_3_ perovskite are believed to be closely linked to the significant pressure dependence of its optical properties. We first traced the evolution of the crystal structure under high pressure using *in situ* synchrotron X-ray diffraction (XRD). Experimental details and analysis methods can be found in the Supplementary Data. Figure S2 shows the selected single-crystal XRD images of MAGeI_3_ collected at ambient pressure, 1.1 GPa and 2.5 GPa. By analyzing the XRD data, two steps of lattice change are observed, an anisotropic variation of the Ge-I bond distances in the low-pressure region followed by a phase transformation at higher pressures.

**Figure 3.**
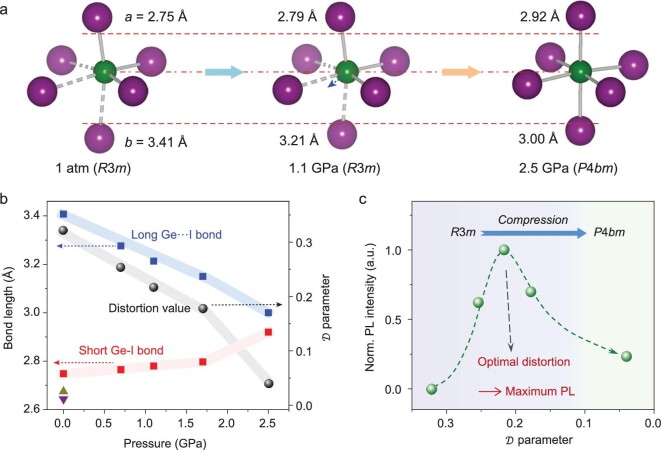
Pressure-induced evolution of the lattice structure in MAGeI_3_. (a) Schematic illustration of the GeI_6_ octahedron at ambient pressure, 1.1 GPa and 2.5 GPa. An anisotropic variation of the Ge-I bond distance in the low-pressure region and a pressure-induced phase transformation in the higher-pressure region are observed. (b) Pressure-dependent variations of the Ge-I bond lengths and $\mathcal{D}$ values, where $\mathcal{D}$ describes the degree of off-centering distortion in the GeI_6_ octahedron. The $\mathcal{D}$ value of MAGeI_3_ is 0.32 at ambient pressure, while those of MASnI_3_ and MAPbI_3_ are 0.03 and 0.01, respectively, plotted as the up and down triangles. The long Ge-I bonds shorten considerably while the short bonds elongate very slightly during compression, which reduces $\mathcal{D}$ to 0.22 at 1.1 GPa. With further pressurization, abrupt changes in both bonds and $\mathcal{D}$ are observed at 2.5 GPa, corresponding to the pressure-induced phase transition from rhombohedral *R*3*m* to tetragonal *P*4*bm*. (c) PL intensity as a function of $\mathcal{D}$. With the decrease of the $\mathcal{D}$ value during compression, the PL strengthens first, reaches the maximum at $\mathcal{D}$ ≈ 0.2, and then weakens.

Upon compression, the long Ge-I bonds in MAGeI_3_ perovskite shorten considerably, whereas the short bonds elongate very slightly (Fig. 3a and b), resulting in an anisotropic variation. That is, the pressure pushes the Ge ion toward the center of GeI_6_ octahedron along the normal direction of the octahedral face, making the octahedron less distorted. Such a Ge centering process reduces the bond-length difference and moves the rhombohedral *R*3*m* structure along a reaction coordinate towards a higher crystalline symmetry at high pressures. At 1.1 GPa, the long and short bond distances change from 3.41 and 2.75 Å to 3.21 and 2.79 Å, respectively, in which their difference is reduced to 15% from the initial value of 24% (Fig. S3). With further pressurization, an abrupt change in both sets of Ge-I bonds is observed at 2.5 GPa, corresponding to the pressure-induced phase transition from rhombohedral *R*3*m* to tetragonal *P*4*bm* (Figs S2 and S4). It is noted that the crystal structure of MAGeI_3_ under high pressure is similar to that of the Sn and Pb perovskites at ambient pressure (Figs 1 and 3a). Upon decompression, the released sample possesses the same crystal structure and lattice constants as the original one (*R*3*m*), as shown in Fig. S5. The variations in XRD peak intensity and width before and after high-pressure treatments indicate the change of orientation and crystallinity, which is due to the pressure-induced phase transition and recrystallization [20,29]. Raman spectra of MAGeI_3_ collected at different pressures confirm the compression-induced structural variations. As the Raman spectra shown in Fig. S6, the peaks weaken and broaden significantly above 2.0 GPa, and the features become similar to that of the Pb perovskites, indicating that the lattice dynamics also become similar [11,36].

To describe the degree of off-centering distortion of the MI_6_ (M = Ge, Sn and Pb) octahedron, we introduce a $\mathcal{D}$ parameter, $\mathcal{D} = \sum\nolimits_{i = 1}^3 {\frac{{| {{a_i} - {b_i}} |}}{{{a_i} + {b_i}}}} $, where *a_i_* and *b_i_* refer to the short and long M-I bond distances, respectively, in one direction (see Supplementary Data for details). The $\mathcal{D}$ value of MAGeI_3_ at ambient pressure is 0.32, over 10 times larger than those of MASnI_3_ and MAPbI_3_ whose values are 0.03 and 0.01, respectively [14]. With increasing pressure, the $\mathcal{D}$ parameter reduces to 0.22 at 1.1 GPa and further drops to 0.04 after transformation to a tetragonal structure at 2.5 GPa (Fig. 3b). The $\mathcal{D}$ value of the high-pressure tetragonal MAGeI_3_ is essentially the same as those of the Sn and Pb iodide perovskites. The PL intensity as a function of the distortion $\mathcal{D}$ is plotted in Fig. 3c. With the decrease of $\mathcal{D}$ during compression, the emission first strengthens significantly, reaches the maximum at $\mathcal{D}$ ≈ 0.2, and then weakens with a further decrease of the distortion. The changing profile suggests that an optimized distortion would lead to the strongest PL in MAGeI_3_. To the best of our knowledge, this behavior has never been observed in other halide perovskites since their distortion cannot be tuned over such a wide range as it can be in MAGeI_3_. The highly distorted GeI_6_ octahedra enable the attainment of an otherwise unexplorable structural region, therefore providing more tuning possibilities for both desired properties and a better understanding of the structure-property relationship.

**Figure 4.**
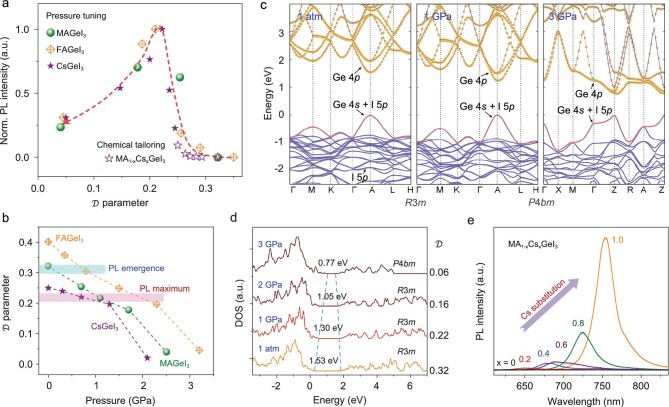
The relationship between emission properties and the off-centering distortion. (a) PL intensity as a function of the distortion $\mathcal{D}$ in the halide perovskites, which describes the pressure-tuned MAGeI_3_ (sphere), FAGeI_3_ (diamond) and CsGeI_3_ (solid star), as well as the chemical-tailored MA_1-x_Cs_x_GeI_3_ (open star). With the decrease of $\mathcal{D}$ during compression, the PL intensity increases first, reaches the maximum at $\mathcal{D}$ ≈ 0.2, and then decreases. The chemical substitution of Cs in MAGeI_3_ follows a similar trend up to the $\mathcal{D}$ value of 0.25 for CsGeI_3_, then pressure further tunes the distortion and enhances the photoluminescence. (b) The pressure-tuned off-centering distortion of MAGeI_3_, FAGeI_3_ and CsGeI_3_. The $\mathcal{D}$ values corresponding to the PL emergence and maximum are consistent for these compounds, which are around 0.3 and 0.2, respectively. (c) The calculated electronic structures of MAGeI_3_ at ambient pressure, 1 GPa and 3 GPa. For the tetragonal phase at high pressure, the direct band gap turns into an indirect type. (d) The calculated density of states (DOS) based on different pressures and distortion $\mathcal{D}$ values. Pressure-induced distortion suppression and lattice contraction together contribute to the band gap narrowing. (e) PL spectra of MA_1-x_Cs_x_GeI_3_ with different Cs concentrations at ambient conditions.

The close relationship between the structural distortion and tunable optical properties can be further demonstrated in the formamidinium germanium iodide (FAGeI_3_) analog, which also crystallizes in a rhombohedral structure while exhibiting an even larger off-centering distortion than MAGeI_3_. The sizeable distortion brings two sets of Ge-I bond distances (2.73 and 3.58 Å) with a difference of 31% in FAGeI_3_ (Fig. S7). From *in situ* XRD measurements, the $\mathcal{D}$ value is determined to be 0.40 at ambient condition and decreases during compression (Fig. S8). FAGeI_3_ also shows no PL signal at ambient conditions, but it can be turned on and tuned by pressure (Fig. S9), similar to MAGeI_3_. The pressure thresholds for the appearance of PL and the attainment of maximum intensity in FAGeI_3_ are 1.0 and 1.9 GPa, respectively, which are higher than the corresponding pressures of 0.3 and 1.1 GPa for MAGeI_3_. This is conceivable considering that the larger formamidinium molecule creates more distorted octahedra in FAGeI_3_. The PL intensity as a function of distortion $\mathcal{D}$ is shown in Fig. 4a (orange diamond shape). In line with the behavior of MAGeI_3_ during compression, the PL emerges and strengthens, reaching the maximum value at $\mathcal{D}$ ≈ 0.2, and weakens thereafter with further decreasing the distortion. The same trend of PL intensity vs. $\mathcal{D}$ value revealed for both MAGeI_3_ and FAGeI_3_ confirms the existence of an optimal distortion which leads to the highest PL efficiency in these halide perovskites. It is worth noting that although the pressures corresponding to the PL appearance and the maximum emission for these two compounds are different, the $\mathcal{D}$ values are consistent at about 0.3 and 0.2, respectively, as shown in Fig. 4b. In addition, the spectacular changes observed in the PL wavelengths as a function of pressure are also reflected in the pressure-dependent absorption spectra (Fig. S10), revealing the profound effects of pressure on the electronic structure. Therefore, off-centering distortion could be an effective order parameter for optimizing the optoelectronic properties of halide perovskites.

The XRD results show that pressure pushes the Ge toward the center of the GeI_6_ octahedron, making the octahedron less distorted. This process suppresses the stereochemical activity of the lone-pair electrons and induces considerable modifications in the conduction and valence bands. Theoretically calculated electronic structures of MAGeI_3_ at ambient pressure, 1 GPa and 3 GPa are shown in Fig. 4c. The band gap narrows significantly under compression, consistent with our experimental observations (Fig. S10). Along with the pressure-induced phase transition, the nature of the band gap turns slightly indirect, which likely contributes to the decreased PL intensity in the higher-pressure region. The calculated density of states (DOS) based on different distortion $\mathcal{D}$ values are given in Fig. 4d and Fig. S11, which suggest that distortion plays a critical role in the electronic structure of MAGeI_3_. By solely considering the gradual suppression of the off-centering distortion (without considering the pressure-induced lattice contraction), the conduction band minimum (CBM) and valence band maximum (VBM) shift synchronously until the $\mathcal{D}$ value drops to 0.2 (Fig. S11). At this stage, the distortion suppression (Ge centering) does not contribute to the band gap narrowing. By pushing the Ge^2+^ more towards the octahedral center, the CBM moves faster than the VBM and this results in the further narrowing of the band gap. Therefore, the highly distorted GeI_6_ octahedron, caused by the large off-centering of Ge^2+^, widens the band gap of the Ge perovskites. This explains the anomaly of the larger-than-expected band gap of MAGeI_3_ (1.9 eV) in comparison to MASnI_3_ (1.3 eV) and MAPbI_3_ (1.6 eV).

In addition to the band structure, defect states could also influence the optical properties of the halide perovskites. Detailed analysis and discussion are given in the Supplementary Data. As a result of the band gap narrowing caused by the pressure-induced distortion suppression and lattice contraction, the harmful trapping states lying in the band gap can be buried into the bands and thus, be deactivated. This argument is schematically illustrated in Fig. S13 and is supported by theoretical simulations (Fig. S14). The compression passivates the trap states and makes the Ge halide perovskite more defect tolerant, which activates the radiative emission. Moreover, the large polarons, forming from charge carriers dressed by long-range lattice deformation in halide perovskites [37–39], also contribute to the change of PL intensity under high pressure. The radiative recombination rate from the energetically stabilized large polarons is reported to be lower than that from free carriers [38], resulting in an inefficient emission from the *e*-*h* polaron states. Upon compression, the reorientational motion of molecular cations slows down and the lattice becomes stiffer. Both trends would lead to the reduced coupling of the motion of organic cations with the deformation of the inorganic framework, giving rise to the destabilization of the large polarons. Pressure changes the dynamic equilibrium between the large polarons and the free carriers, which partially contributes to the enhanced PL of the hybrid perovskites. Therefore, the observed variations of the PL property can be elucidated by comprehensively considering the contributions from the pressure-induced distortion suppression, trap state deactivation, polaron destabilization and phase transition.

Based on our understanding of the underlying mechanisms of the dramatic pressure-induced changes in Ge halide perovskites, we purposefully substituted the MA^+^ ions in MAGeI_3_ with smaller Cs^+^ to simulate the pressure effects. This smaller A-cation substitution would lower the Ge^2+^ off-centering level and reduce the structural distortion in the halide perovskites. Scanning electron microscopy images and the corresponding energy dispersive spectroscopy mappings of the Cs-substituted samples are shown in Fig. S15, which demonstrate the uniform distribution of Cs. PL measurements were performed on the MA_1-x_Cs_x_GeI_3_ samples with Cs content x = 0, 0.2, 0.4, 0.6, 0.8 and 1. Impressively, the PL can be induced by Cs substitution, and the emission intensity increases with the Cs content, as shown in Fig. 4e. The distortion $\mathcal{D}$ of these materials decreases from 0.32 to 0.25 as Cs increase from 0 to 1 (Fig. S16), as determined by XRD measurements. With the decrease of the $\mathcal{D}$ value by Cs substitution, the PL intensity of MA_1-x_Cs_x_GeI_3_ increases, which complies with the principle uncovered by the high-pressure experiments, shown as the open-star points in Fig. 4a. Coincidentally, a parabolic trend of the optical band gap versus the organic A cation size has been revealed recently in Ruddlesden-Popper perovskites, which can be attributed to the changes of chemical pressure applying to the inorganic framework [40].

The chemical substitution triggers and enhances the PL by suppressing the distortion, yet has not reached the optimal level according to the newly discovered structure-property relationship. For this reason, high-pressure experiments were performed on CsGeI_3_, whose $\mathcal{D}$ value is 0.25, to further tune the off-centering distortion towards the maximum PL. As shown in Fig. S17, the emission increases significantly with increasing pressure, reaching the peak value at 0.7 GPa. Notably, the PL intensity of CsGeI_3_ is enhanced by more than ten times under high pressure in comparison to its initial value at ambient conditions. Similar to MAGeI_3_ and FAGeI_3_, the $\mathcal{D}$ value of CsGeI_3_ decreases during compression (Fig. S18). The relationship between the PL intensity and the tunable distortion $\mathcal{D}$ of all these halide perovskites collapses on the same curve, as shown in Fig. 4a, where the experimental data of CsGeI_3_ are plotted as solid stars. Specifically, the pressure-regulated distortion maximizes PL when $\mathcal{D}$ reduces to around 0.2 during compression, following the exact same trend as revealed in MAGeI_3_ and FAGeI_3_. Although the pressures corresponding to the brightest PL for these halide perovskites are different, the optimal distortion $\mathcal{D}$ value is always around 0.2, as summarized in Fig. 4b. Therefore, the distortion is a suitable variable that can be regulated for the desired properties of halide perovskites.

## CONCLUSION

By regulating the octahedral distortion in halide perovskites using pressure engineering and chemical substitution, we have reached an otherwise unexplorable structural region for tuning and probing properties. Compression induces strong photoluminescence with impressive tunability of both emission intensity and wavelength in the highly distorted germanium halide perovskites. For both MAGeI_3_ and FAGeI_3_, the PL intensity shows a 20-fold boost within a 1 GPa increase in pressure; the emission wavelength exhibits a pressure dependence of over 180 nm/GPa, 4–5 times higher than reported values of other halide perovskites. The *in situ* high-pressure probes, in combination with first-principles calculations, reveal a universal relationship between the off-centering distortion and PL property of halide perovskites, and demonstrate that regulating the distortion degree $\mathcal{D}$ towards 0.2 leads to the brightest emission. Applying this principle as a guideline, PL can be induced in MA_1-x_Cs_x_GeI_3_ by chemical substitution using the smaller sized Cs^+^, which decreases the off-centering distortion, acting in a similar role to external pressures. The compression of a fully substituted sample of CsGeI_3_ further regulates the distortion to the optimal value at 0.7 GPa, which maximizes the PL intensity with a 10-fold increase. Our findings lay the groundwork for the fundamental understanding of the structure-property relationship in halide perovskites and open new paths for materials design and optimization by leveraging their distortion degree of freedom.

## Supplementary Material

nwaa288_Supplemental_File
