# Supplementary material for: Regulating off-centering distortion maximizes photoluminescence in halide perovskites
Source: Natl Sci Rev. 2020 Nov 30;8(9):nwaa288. doi: 10.1093/nsr/nwaa288 (PMC8433095; doi:10.1093/nsr/nwaa288)
Supplement: nwaa288_Supplemental_File [file nwaa288_supplemental_file.docx]

Supplementary Data

**Regulating off-centering distortion maximizes photoluminescence in halide perovskites**

Xujie Lü^1*^, Constantinos Stoumpos^2,3^, Qingyang Hu^1^, Xuedan Ma^4^, Dongzhou Zhang^5^, Songhao Guo^1^, Xiaofeng Guo^6^, Justin Hoffman^2^, Kejun Bu^1^, Yingqi Wang^1^, Cheng Ji^1^, Haijie Chen^2^, Hongwu Xu^7^, Quanxi Jia^8^, Wenge Yang^1^, Mercouri G. Kanatzidis^2*^, and Ho-Kwang Mao^1^

^1^ *Center for High Pressure Science and Technology Advanced Research (HPSTAR), Shanghai 201203, China.*

^2^ *Department of Chemistry, Northwestern University, Evanston, IL 60208, USA.*

^3^ *Department of Materials Science and Technology, Voutes Campus, University of Crete, Heraklion GR-70013, Greece.*

^4^ *Center for Nanoscale Materials, Argonne National Laboratory, Lemont, IL 60439, USA.*

^5^ *Partnership for Extreme Crystallography, University of Hawaii at Manoa, Honolulu, HI 96822, USA.*

^6^ *Department of Chemistry and Alexandra Navrotsky Institute for Experimental Thermodynamics, Washington State University, Pullman, WA 99164, USA.*

^7^ *Earth and Environmental Sciences Division, Los Alamos National Laboratory, Los Alamos, NM 87545, USA.*

^8^ *Department of Materials Design and Innovation, University at Buffalo – The State University of New York, Buffalo, NY 14260, USA.*

*e-mail: xujie.lu@hpstar.ac.cn; m-kanatzidis@northwestern.edu

**Experimental details**

***Sample preparation and structural description***

The synthesis of CH_3_NH_3_GeI_3_ (hereafter MAGeI_3_) crystals has been described in detail in previous work.[1] A 2-neck flask was charged with a mixture of HI (6.8 mL, 7.58 M) and H_3_PO_2_ (3.4 mL, 9.14 M) solutions. GeI_4_ (580 mg, 1 mmol) was dissolved in the mixture upon heating the flask to 120 °C using an oil bath, under constant magnetic stirring, forming a bright yellow solution. The direct addition of GeO_2_ (105 mg, 1 mmol) in the solvent mixture still produces the bright yellow solution after ∼30 min. A stoichiometric amount of CH_3_NH_3_I (159 mg, 1 mmol) was added to the solution. The solution was evaporated to approximately half its original volume by heating at 120 °C. The stirring was discontinued, and the solution was left to cool down to room temperature. Upon cooling, deep-red crystals of CH_3_NH_3_GeI_3_ were precipitated. The crystals were left to grow inside the mother liquor for 24 h under a nitrogen atmosphere before being filtered and washed with the minimum amount of degassed ethanol. A similar method was used for the synthesis of formamidinium germanium iodide and the cesium substituted MAGeI_3_. At ambient conditions, different from lead and tin perovskites, germanium halide perovskites crystallize in a rhombohedral structure of the polar *R*3*m* space group (Fig. 1). The corner-sharing [GeI_6_]^4–^ octahedra form an infinite three-dimensional (3D) framework through Ge–I–Ge bridges. The trigonal distortion causes loss of the 4-fold symmetry axes present in the tetragonal and cubic perovskites.

***In situ high-pressure structural characterizations***

Symmetric diamond anvil cells (DACs) were employed to generate high pressures. A rhenium gasket was pre-indented to about 40 µm in thickness followed by laser-drilling a 200 µm diameter hole in the central part to serve as the sample chamber. Germanium halide perovskite crystals and ruby microspheres (for pressure measurements) were loaded into the chamber. Neon gas was loaded at GeoSoilEnviroCARS (Sector 13 at Advanced Photon Source, Argonne National Laboratory) to serve as the pressure-transmitting medium. The pressures were determined by the ruby fluorescence method.

The *in situ* single crystal X-ray diffractions at high pressures were carried out at the experimental station 13 BM-C of the Advanced Photon Source (APS), Argonne National Laboratory (ANL).[2] The X-ray beam was monochromated with silicon 311 crystal to 28.6 keV (0.434 Å), with 1 eV bandwidth. A Kirkpatrick-Baez mirror system was used to obtain a vertical × horizontal focus spot size of 15 μm × 15 μm, measured as the full width at half maximum (FWHM). The MAR165 Charge Coupled Device (CCD) detector (Rayonix) was placed about 160 mm away from the sample, and the ambient LaB_6_ powder was used to calibrate the distance and tilting of the detector. The sample was placed on the rotation center of the diffractometer, and was aligned by an optical microscope. For high pressure diffraction, the angular range of the wide rotation exposure was φ=-30° to φ=30°, limited by the maximum opening angle of the diamond anvil cell (60º), and followed by a series of step φ-exposures, each covering 1° scan width. The typical exposure time was 2 s/°. At zero diffractometer position, the φ scan rotation axis for the 13BM-C diffractometer is in the horizontal plane of the instrument, and is perpendicular to the incident X-ray direction. After the first set of wide and step φ exposures, collected at zero detector position, similar data were collected with the detector rotated about the horizontal axis (2θ) by 20°, and then with detector rotated about the vertical axis (ν) by ±10°. The wide rotation exposures were used to extract *d*-spacings, azimuthal angles around the beam center, and peak intensities of each diffraction peak, and the step φ exposures provided the third spatial coordinate necessary for reconstructing the crystal’s reciprocal lattice and were used to index the diffraction pattern.

The diffraction images were analyzed using the ATREX/RSV software package.[3] Polarization, Lorentz, and empirically determined diamond absorption corrections were applied to the fit peaks. The unit cell and orientation matrix were determined in RSV for each dataset. Lattice parameters were refined in RSV using a least-squares fitting procedure. Corrected peak intensities were used to refine the crystal structures with SHELXL software, facilitated by Olex2 general user interface.[4-5] The VESTA software was used to calculate the chemical bonding and polyhedral volumes.[6]

Since the crystal could be destroyed by the pressure-induced structural transition at above 2 GPa, the quality of XRD data was not good enough to analyze using the single-crystal refining method alone. To this end, we further conducted the powder XRD measurements for MAGeI_3_ at 16 BM-D station of the High-Pressure Collaborative Access Team (HPCAT) at APS, ANL. The XRD data were analyzed using the Rietveld method with the General Structure Analysis System (GSAS) program.[7] The starting structural parameters of the rhombohedral (*R3m*) phase were taken from our single-crystal XRD results, which is consistent with the study of MAGeI_3_ by Kanatzidis *et al*.[1] We then used the refined structural parameters as the starting parameters for the next pressure and continued this procedure systematically with increasing pressure. The Rietveld analysis of the XRD collected at 2.5 GPa is shown in Fig. S4. The same experimental and analytical methods were used for the FAGeI_3_ compound.

***In situ high-pressure optical measurements***

Since the optical properties of halide perovskites have been reported to be strongly dependent on many factors such as crystal size, grain boundaries, and concentration of defects,[8-9] MAGeI_3_ and FAGeI_3_ single crystals were used in our experiments. For pressure-dependent optical measurements, the samples were loaded into DACs and neon was used to serve as the pressure-transmitting medium. Then the DACs were loaded onto a home-built confocal laser microscope. Excitation beams from a pulsed laser with a wavelength of 400 nm was focused onto the samples by a long-working-distance microscope objective (10×, NA = 0.30). Photoluminescence from the samples was collected by the same objective and sent to detectors. A charge-coupled device and a two-dimensional InGaAs array camera mounted on 300 mm spectrographs were used to take photoluminescence images and spectra in the visible and near-IR wavelength ranges, respectively. The PL intensity is determined by integrating the peak areas. The Raman spectra were collected by a home-built system at GSECARS, Advanced Photon Source, Argonne National Laboratory. Different laser energies of 532 nm, 660 nm, and 946 nm were used in order to avoid the fluorescence background. The UV-Vis-NIR absorption spectra under high pressures were collected by a home-designed optical system (assembled by Ideaoptics).

***Theoretical Calculations***

First-principles calculations were performed in the framework of density functional theory through the Vienna *ab initio* simulation package[10]_._ The exchange-correlation functions are described by the Generalized Gradient Approximation (GGA) under the Perdew-Burke-Ernzerhof parameterization revised for soilds (PBEsol)[11-12]. Projected-augmented wave potentials model 14 valence electrons for Ge (3*d*^10^4*s*^2^4*p*^2^), 7 for I (5*s*^2^5*p*^2^), 4 for C atoms (2*s*^2^2*p*^2^), 5 for N atoms (2*s*^2^2*p*^3^), and 1 for H (1*s*^1^). We used a plane-wave basis set with kinetic 550 eV energy cut off that is sufficient to optimize the structure until forces acting on each atom are less than 0.02 eV Å^-1^. We employed Monkhorst mesh of 5×5×5 *k* points for *R*3*m* phase and 3×3×4 *k* points for the high-pressure *P*4*bm* phase. Crystal structures for simulation are the same as the ones resolved from in situ X-ray diffraction experiments. In cases of the resolved structure lacking positions of H, those H atoms were added according to the charge balance of the organic part. Spin-orbit coupling effects were turned on to calculate density of states and band structures[13]. The band structures were sampled along the high-symmetry points in the Brillouin zone according to the work by Setyawan and Curtarolo[14]. The methods we used for the calculations have been widely employed to simulate the structural and electronic properties of multiple halide perovskites[15-22].

Simulations mainly help understand two points: the effect of the off-centering distortion on the band gaps and the electronic structures for the defected MAGeI_3_. For the off-centering effect, we performed a selective dynamics calculation such that atoms in the Ge-I octahedron are fixed in certain distortion while the rest of atoms are allowed to relax[23-24]. We engineered the structures with the octahedron in MAGeI_3_ varying distortion parameter 𝒟 values from 0.06 to 0.32 in this study. The whole structure is fully relaxed at target pressures of 1 atm, 1 GPa, and 2 GPa. In Fig. S11, we observed the distortion parameter 𝒟 has a profound effect on the electronic structure of MAGeI_3_. A consistent broadening of band gap width is observed for all calculated pressures with an increasing level of distortion up to 𝒟 = 0.19, above which the broadening effect flat out. A sudden reduction of 𝒟 was observed during the transition from the rhombohedral phase to the tetragonal phase. The predicted band-gap narrowing is well consistent with the experimental results.

For the electronic structures of defected MAGeI_3_, a 2×2×2 supercell was constructed for this calculation, and one I or MA component is removed to generate the defect[25]. All variables in the crystal are allowed to relax such that atoms can move towards to the defect site. Our results suggested that intrinsic defects such as I and MA vacancies create trap states in the band gap (Fig. S12). Those trap states could be deactivated at higher pressures, as shown in Fig. S14.

**Detailed Discussion**

***Raman spectroscopy under high pressures***

Raman spectra of MAGeI_3_ at various pressures during compression and decompression were recorded in the low-frequency range with the aim of gaining further insight on the local symmetry evolution. For the analysis of the vibrational modes, the halide perovskite was treated as interacting [GeI_3_]^−^ pyramid rather than [GeI_6_]^4−^ octahedron (Fig. S6a). Such a configuration includes four Raman-active modes at ambient condition, that is, ν_1_ and ν_3_, which are the stretching modes, and ν_2_ and ν_4_, which are the bending modes. The stretching modes soften with increasing pressure due to the anisotropic variation of Ge-I bond distance in the low-pressure region before phase transition. As can be seen in Fig. S6b, the Raman spectrum peaks broaden during compression. At above 2.0 GPa, the peaks weaken and broaden significantly, and their features are similar to the spectra of Pb perovskites, indicating that the lattice dynamics also become similar[26-27]. This change corresponds to the pressure-induced phase transformation from rhombohedral (in GeI_3_ pyramids) to tetragonal (in GeI_6_ octahedra) at above 2 GPa, which is consistent with the XRD results.

***Distortion 𝒟 parameter of the halide perovskites***

The 𝒟 parameter, $\mathcal{D}=\sum_{i=1}^{3} \frac{|a_{i}-b_{i}|}{a_{i}+b_{i}}$, is introduced to describe the degree of the off-centering distortion in MI_6_ (M = Ge, Sn, and Pb) octahedron in the perovskite structure, where *a_i_* and *b_i_* refer to the short and long M-I bond distances, respectively, in one direction. In MAGeI_3_ perovskite, Ge sits away from the octahedral center along all the three directions of I-Ge-I bonds (Fig. 1 and Fig. S3). This off-centering distortion brings two sets of Ge-I bond distances of 2.75Å and 3.41 Å, showing a huge difference of 24%. For comparison, both MAPbI_3_ and MASnI_3_ perovskites crystallize in the tetragonal structure at ambient conditions, where the octahedral distortion is only from the shift of metallic cation along one direction in the octahedron. By using the 𝒟 parameter, one can quantitively compare the degree of octahedral distortion in the halide perovskites. At ambient conditions, the 𝒟 value of MAGeI_3_ is determined to be 0.32, while those of Sn and Pb halide perovskites are only 0.03 and 0.01,[28] respectively. Formamidinium germanium iodide (FAGeI_3_) possesses even higher GeI_6_ off-centering distortion, brings two sets of Ge-I bonds with a large difference (Fig. S7). The length of three short bonds is 2.73 Å and that of the three long bonds is 3.58 Å, whose difference reaches 31% and the 𝒟 value of FAGeI_3_ is determined to be 0.40 at ambient condition.

In order to expand the proposed relationship and cover more general situations of octahedral distortion (not just the isotropic off-centering in the Ge perovskites), we further modify the equation for determining the distortion parameter as $D=\sum_{i=1}^{6} \frac{{|d}_{i}-d|}{2d}$, where *d* is the mean M-X bond distance and *d_i_* are the six individual bond distances in the perovskite structure. As shown in Fig. S19, we have added some cases of temperature effect from the literature as well as the pressure effect. We analyzed these PL and structural results in terms of the *D* parameter and found that the PL-*D* relationship complies well with the principle uncovered in this work. This further supports and expands the proposed relationship, where regulating the octahedral distortion can be used to improve the PL performance in the halide perovskites.

***Influence of intrinsic defects on the optoelectronic properties under high pressure***

Intrinsic atomic defects in semiconductors act as the trap sites, which would weaken or even quench the photoluminescence due to the nonradiative recombination.[25, 29-31] The major intrinsic defects in halide perovskites have demonstrated to be point defects including A-site and halide vacancies as well as Schottky-type vacancies, due to their low formation energy.[32] Our density functional theory (DFT) calculations uncovered that intrinsic defects in MAGeI_3_ such as I and MA vacancies form defect states in the band gap, while the Schottky-type vacancies form defect states within the band. As shown in Fig. S12, I vacancies form an interband state of ~90 meV away from the conduction band edge and the MA vacancies form a state of ~50 meV away from the valence band edge, much deeper than the defect states in MAPbI_3_ which is below 20 meV.[33-36] Due to the dynamically flexible and soft lattices in the halide perovskites, the pressure-induced changes in electronic structures are dramatic. With the increase of external pressure, the defective states would be buried into the band, as evidenced by the DFT calculations (Fig. S14). Therefore, compression would deactivate the trap states and make the Ge halide perovskites more defect-tolerant and thus enhance their optical properties, as illustrated in Fig. S13.

**Supplementary Figures**


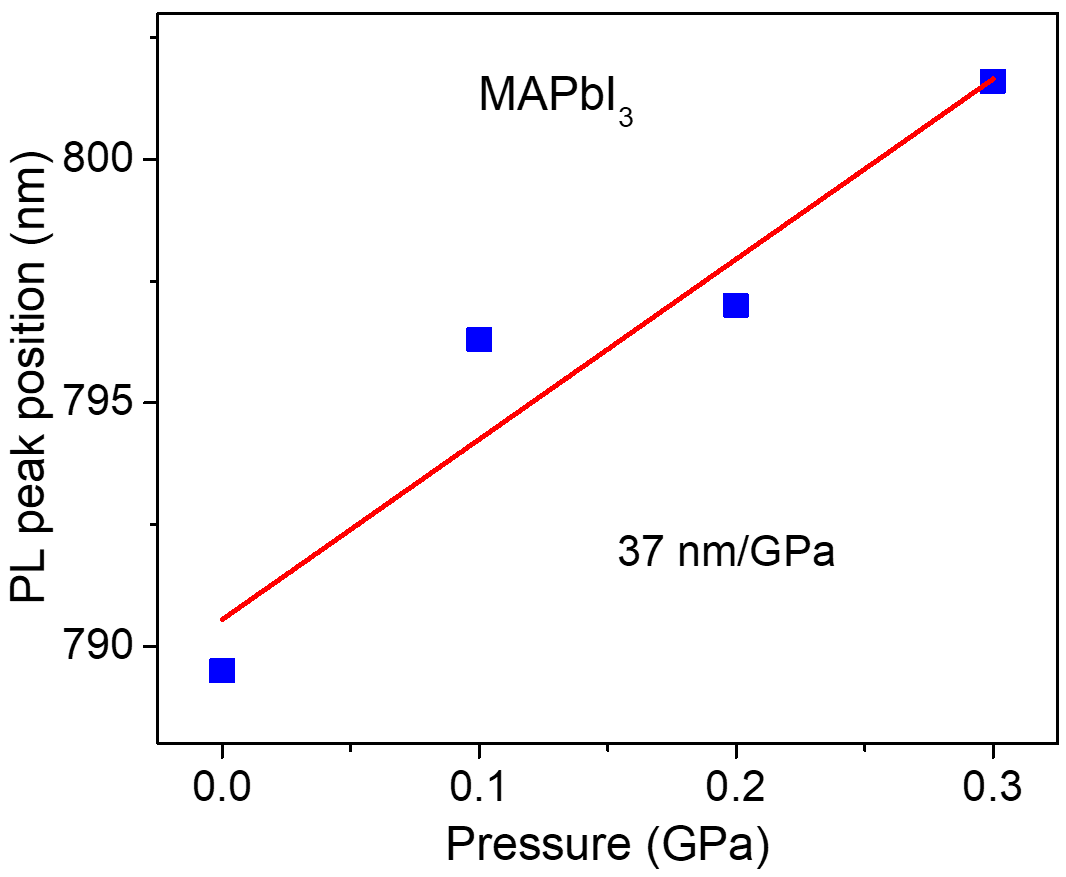


**Fig. S1 |** Pressure-dependent PL peak positions of CH_3_NH_3_PbI_3_, which shows a tunability of 37 nm/GPa. The results are reproduced from Ref. [[37]].


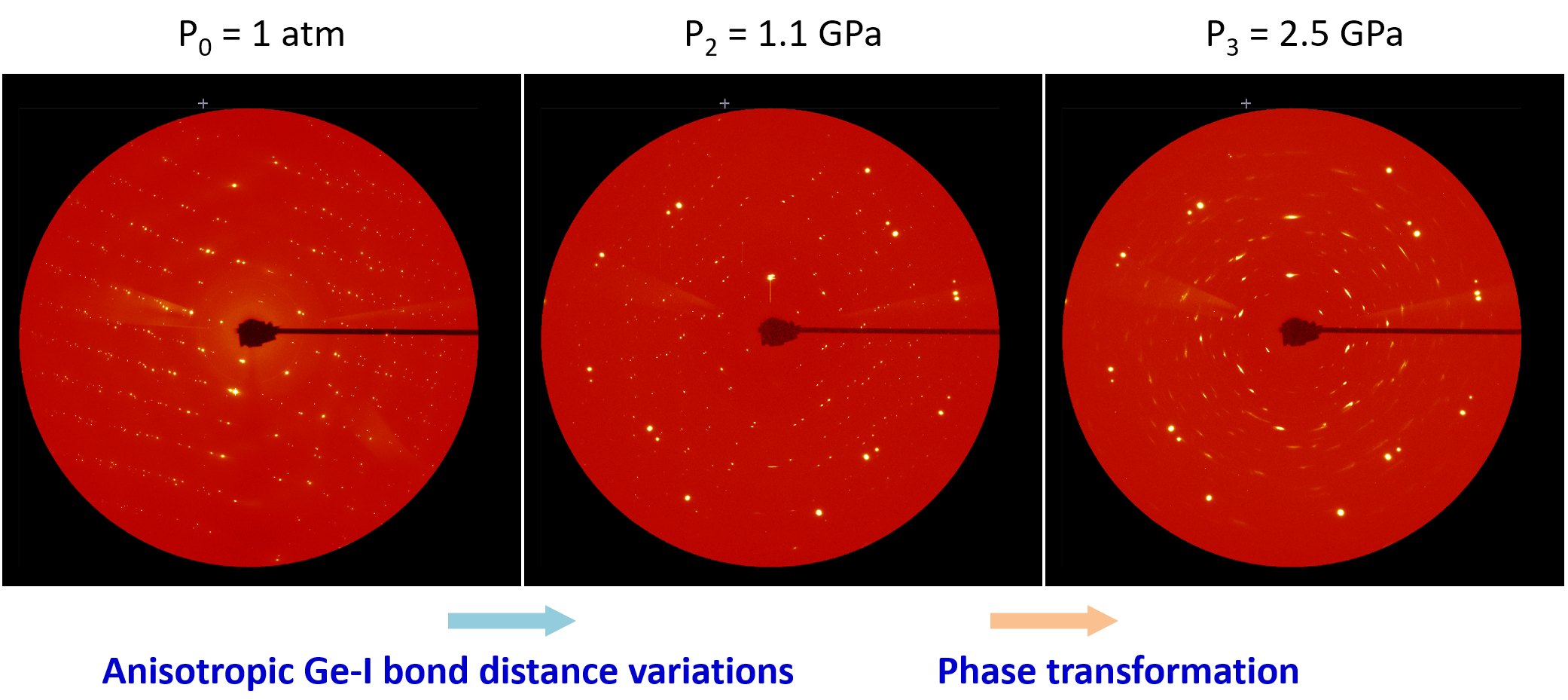


**Fig. S2 |** Selected single-crystal XRD images collected at ambient pressure, 1.1 GPa, and 2.5 GPa, respectively. An anisotropic variation of Ge-I bond distance in low-pressure range and a pressure-induced phase transformation in the higher region are observed.


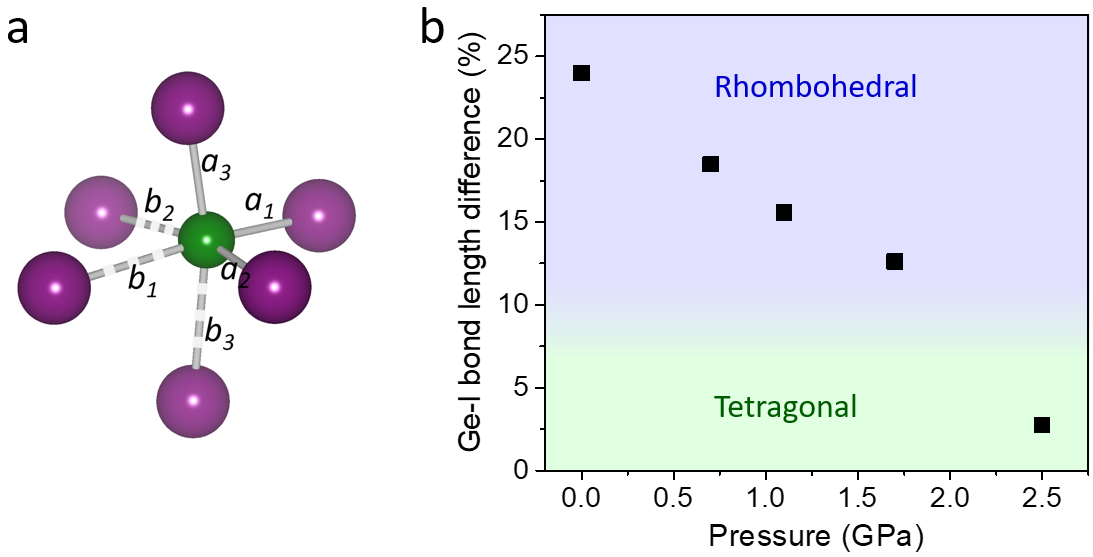


**Fig. S3 |** **a**, GeI_6_ octahedral structure, *a_i_* and *b_i_* refer to the short and long Ge-I bond distances, respectively. **b**, The difference of long and short Ge-I bond lengths in MAGeI_3_ as a function of pressure. During compression, the long Ge-I bonds shorten considerably while the short bonds elongate slightly, which lowers the difference of bond length from 24% at ambient pressure to 15% at 1.1 GPa. With further pressurization, an abrupt change is observed at 2.5 GPa, corresponding to the pressure-induced phase transition from rhombohedral *R*3*m* to tetragonal *P*4*bm*.


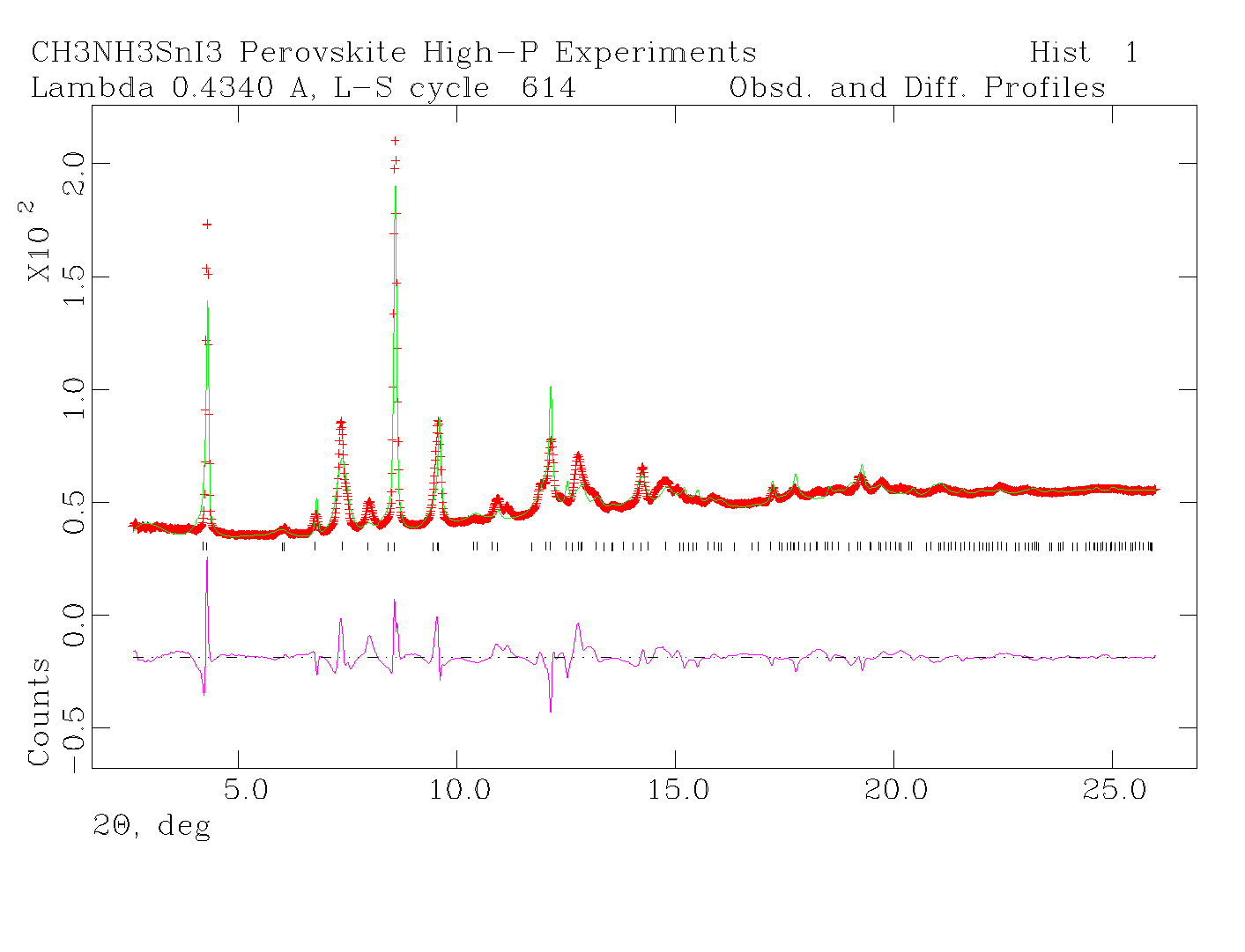


**Fig. S4 |** Rietveld analysis of the powder XRD data of MAGeI_3_ at 2.5 GPa with the *P*4*bm* structure, *a* = 8.207 Å, *c* = 5.911 Å, and V = 398.112 Å^3^. No attempts were made to refine the MA^+^ position due to the limited resolution of the high-pressure XRD data.


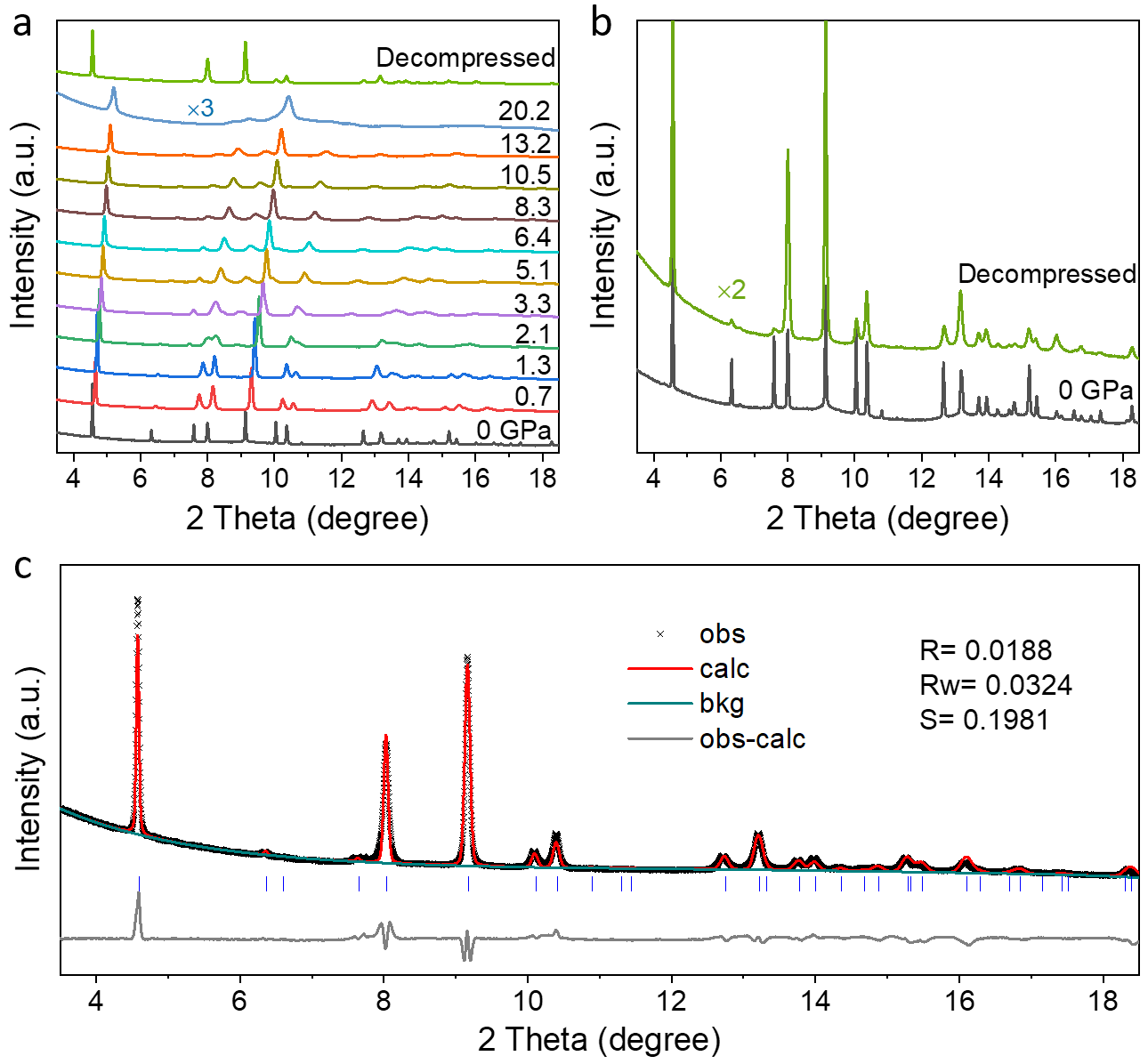


**Fig. S5 |** **a**, XRD patterns of MAGeI_3_ perovskite under high pressures. **b**, Comparison of the XRD patterns of the sample before and after pressure treatment, from where changes in grain orientation and crystallinity can be observed. **c**, Rietveld analysis of the powder XRD data of MAGeI_3_ after high-pressure treatment. The structure crystallizes in a *R*3*m* structure, the same as the original phase.


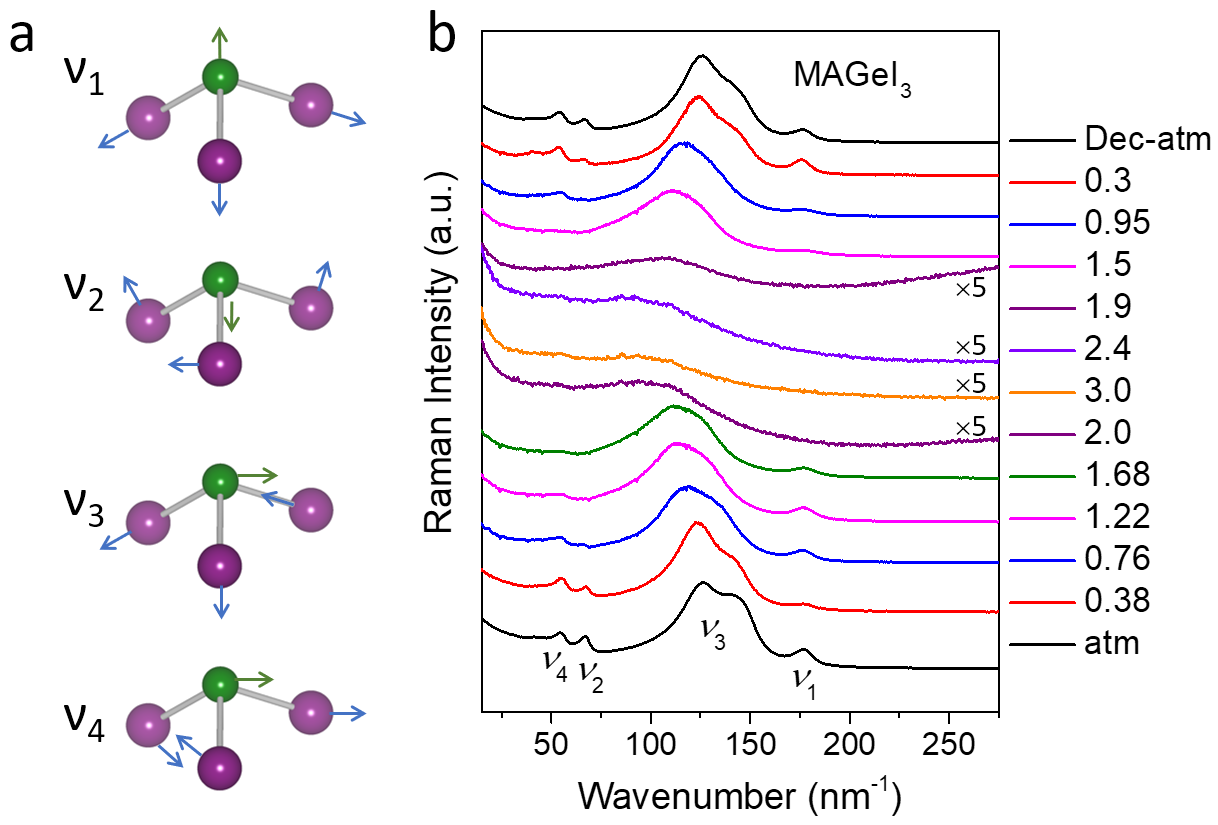


**Fig. S6 | a**, A schematic of the active vibrational Raman modes of the [GeI_3_]^−^ pyramids. **b**, In situ Raman spectra of MAGeI_3_ during compression and decompression.


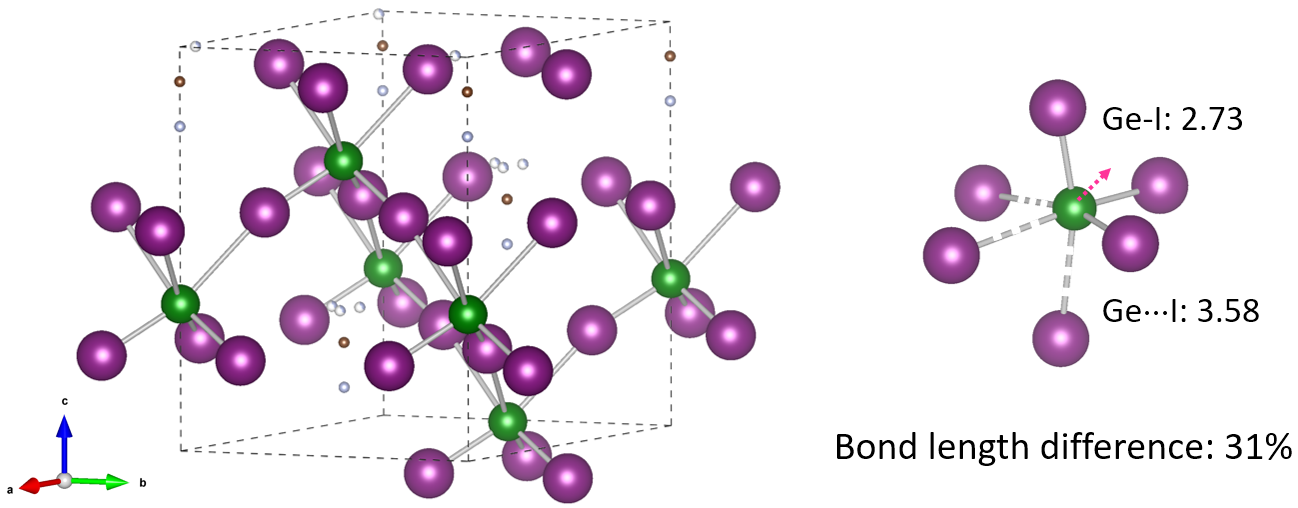


**Fig. S7 |** **Crystal structure of** **FAGeI_3_.** It crystallizes in a rhombohedral structure *R*3*m* and the large distortion is caused by the shift of Ge along all the three directions of I-Ge-I bonds in GeI_6_ octahedron (*i.e.* along the normal direction of the octahedral face). Such a off-centering distortion brings two sets of Ge-I bonds: the length of three short bonds is 2.73 Å and that of the three long bonds is 3.58 Å. Their bond length difference reaches 31%, even higher than that of MAGeI_3_.


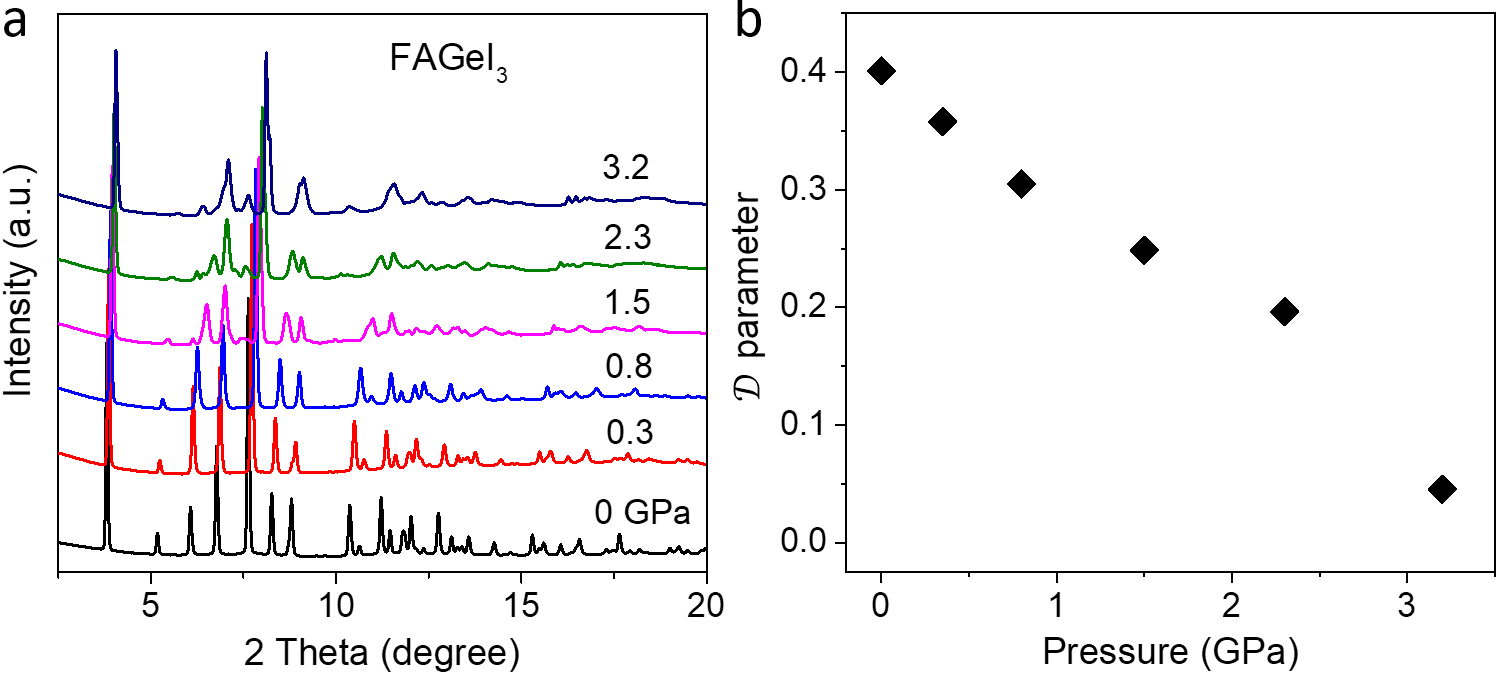


**Fig. S8 |** **a**, Integrated XRD patterns of FAGeI_3_ perovskite under high pressures. **b**, Distortion 𝒟 parameter of FAGeI_3_ as a function of pressure. 𝒟 value of FAGeI_3_ is determined to be 0.40 at ambient pressure, and decreases significantly with pressure increasing.


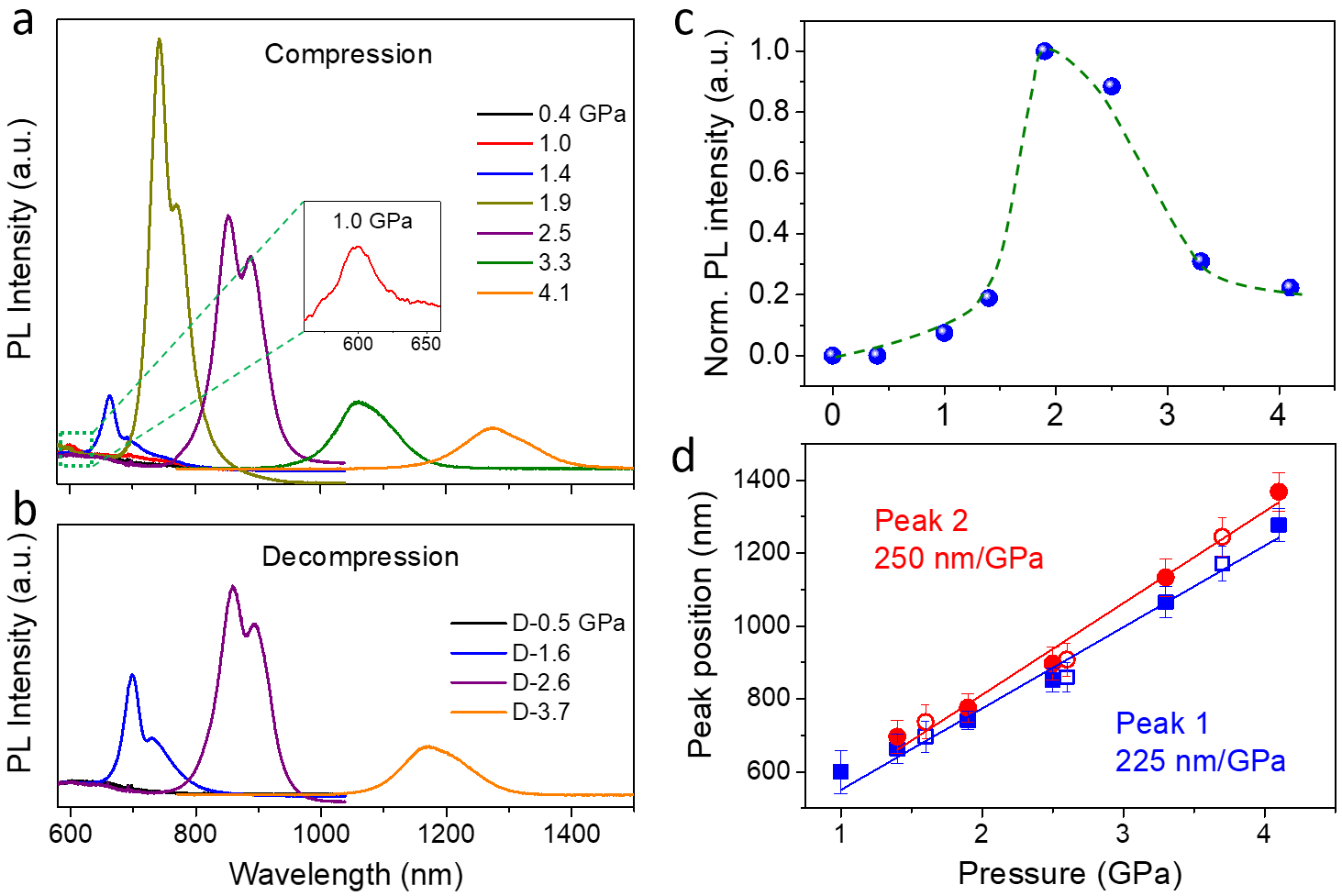


**Fig. S9 |** **Pressure-dependent photoluminescence properties of FAGeI_3_.** In situ PL spectra under high pressure during compression (**a**) and decompression (**b**). The insets show the zoomed-in PL spectrum at 1.0 GPa. **c**, Pressure dependence of spectrally integrated PL emission intensity. The PL appears at around 1 GPa and reaches the maximum value at around 2 GPa. **d**, PL peak position in response to pressure which shows a super wide turnability of over 220 nm/GPa, even higher than that of MAGeI_3_.


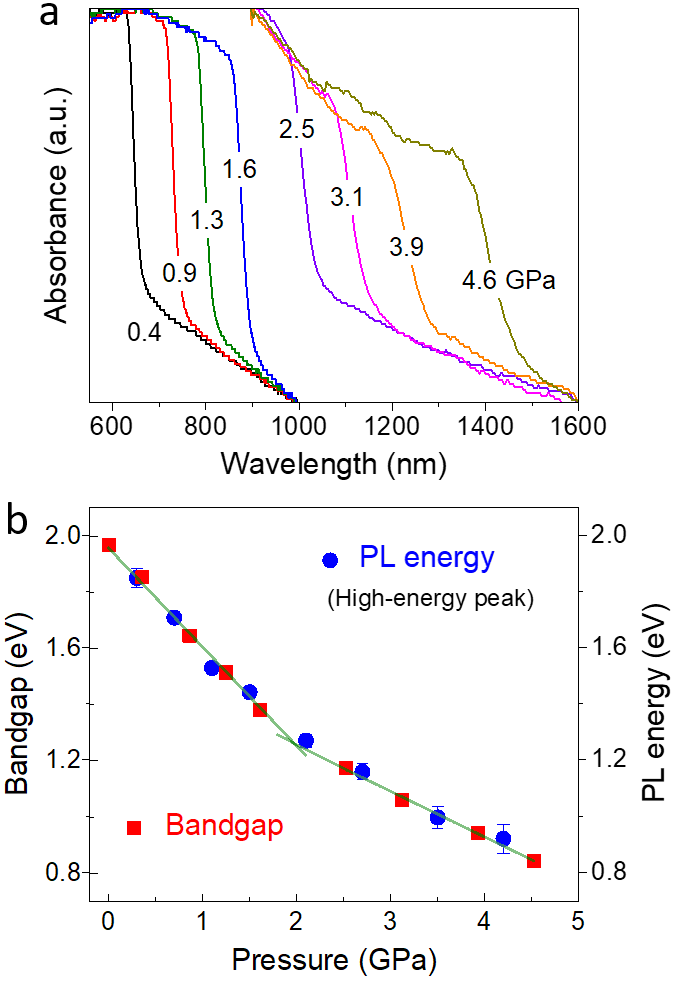


**Fig. S10 |** **Pressure-induced band gap narrowing of MAGeI_3_.** **a**, UV-Vis-NIR absorption spectra under high pressures. **b**, Band gap evolution in comparison with the PL emission energy. The band gaps determined from the absorption edges are almost the same as those determined from the high-energy PL emission peak.


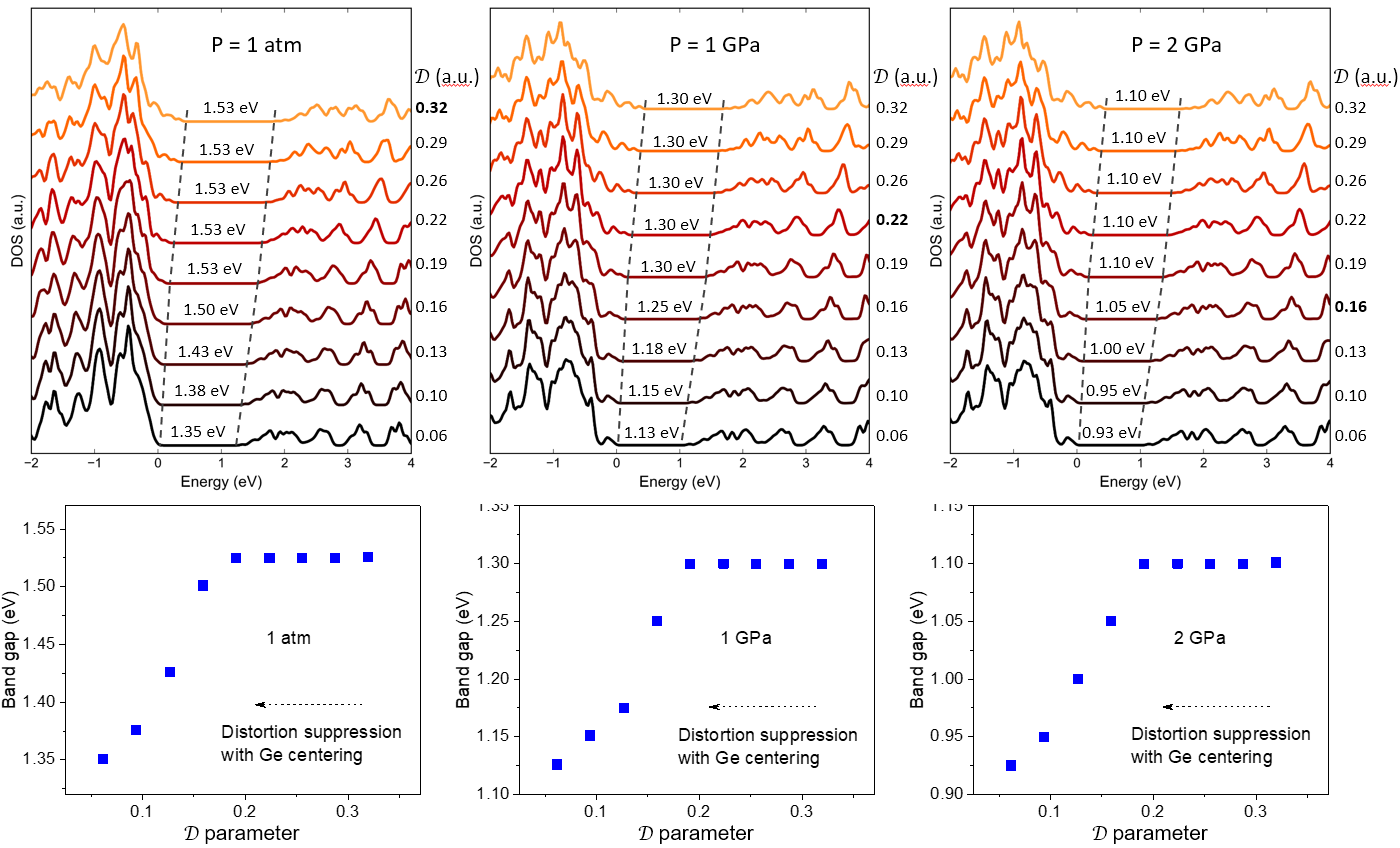


**Fig. S11 |** **Evolution of the density of states and band gap by tuning the off-centering distortion of GeI_6_ octahedron as parameterized by the value of 𝒟.** Conduction band minimum (CBM) and valence band maximum (VBM) shift parallelly first down to a 𝒟 value of 0.19. With further pushing Ge towards the octahedral center, CBM moves fast than VBM, contributing to the narrowing of the band gap in addition to lattice shrinkage.


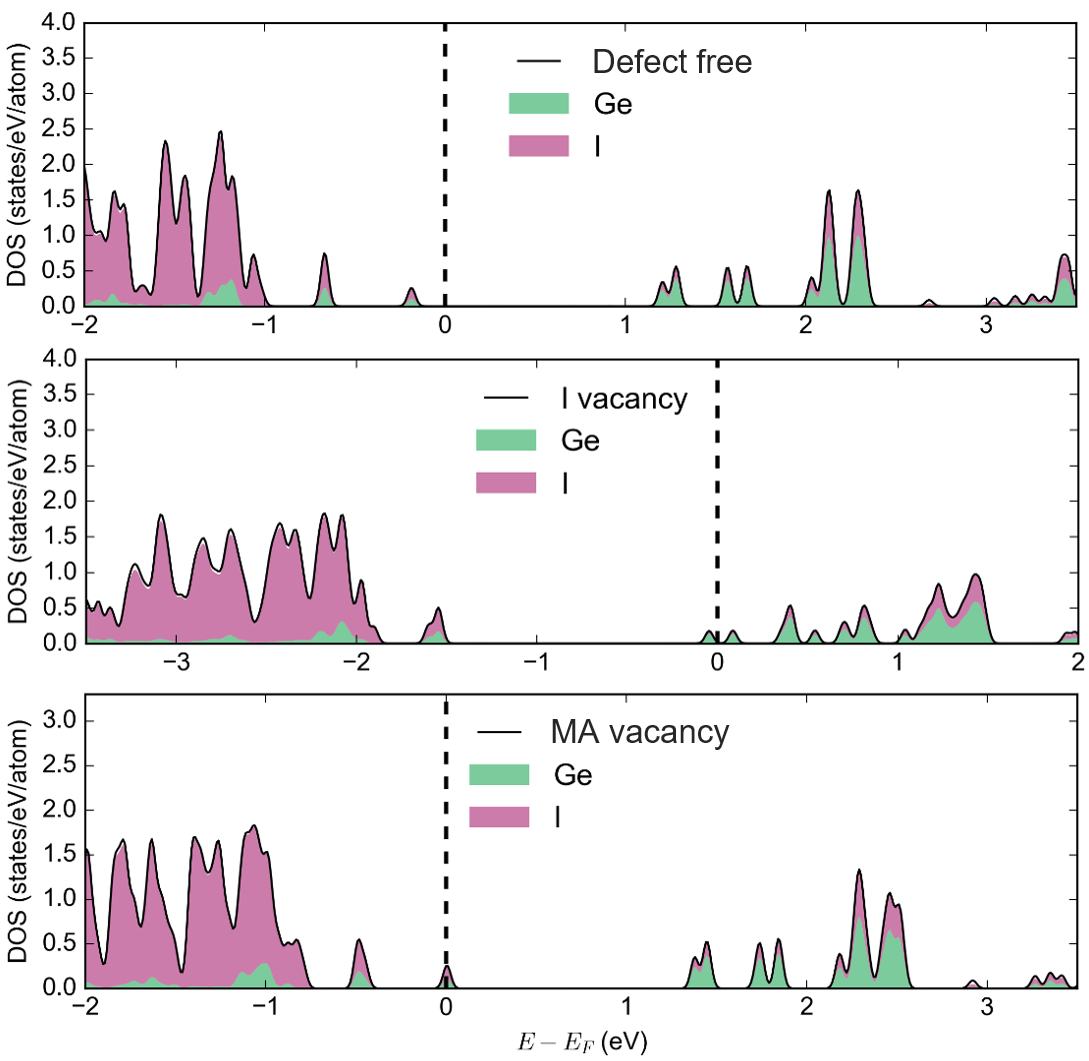


**Fig. S12 |** Density of states (DOS) of defect-free MAGeI_3_ in comparison with the systems with I and MA vacancies. The I vacancies generate the defect level near the conduction band, while MA vacancies form the energy levels near the valence band.


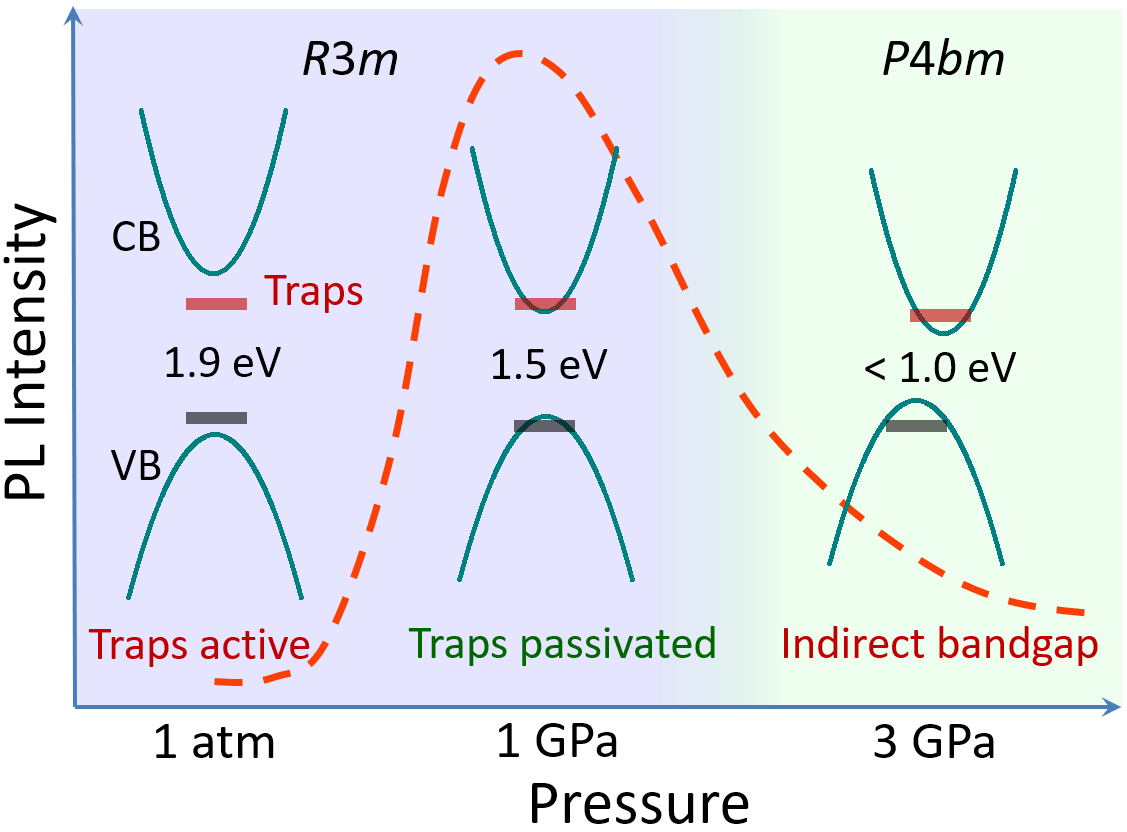


**Fig. S13 | Schematic illustration of the mechanisms of pressure-dependent PL property.** Under compression, the pressure-induced distortion suppression and energy-band broadening would passivate the intrinsic trap states and make it more defect-tolerant, which activates the radiative emission. The pressure-dependent off-centering distortion further tunes and maximizes the PL. In the higher pressure region, a direct-to-indirect band gap transition occurs due to the structural transformation from rhombohedral to tetragonal, giving rise to the decreased emission intensity.


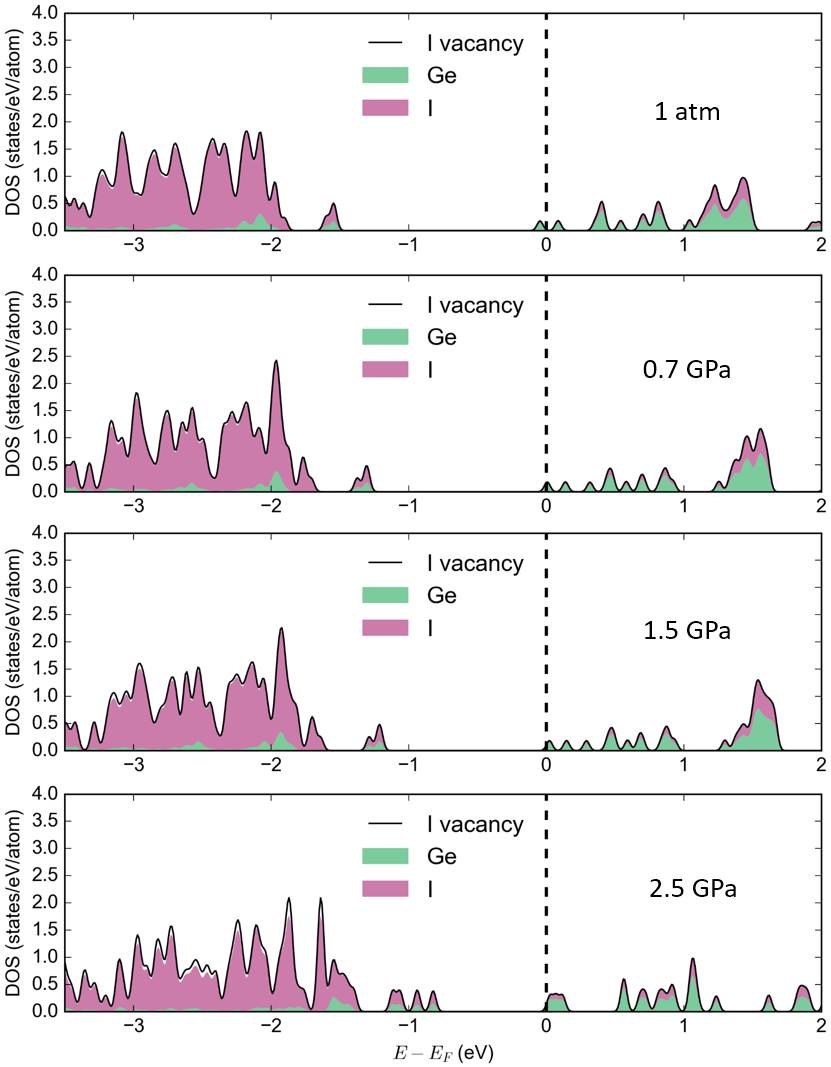


**Fig. S14** **| DFT calculations for the variation of defective states of I vacancies during compression.** Under compression, the defective states would be buried into the band. Therefore, compression would deactivate the trap states and make the Ge halide perovskites more defect-tolerant and thus enhance the optical properties.


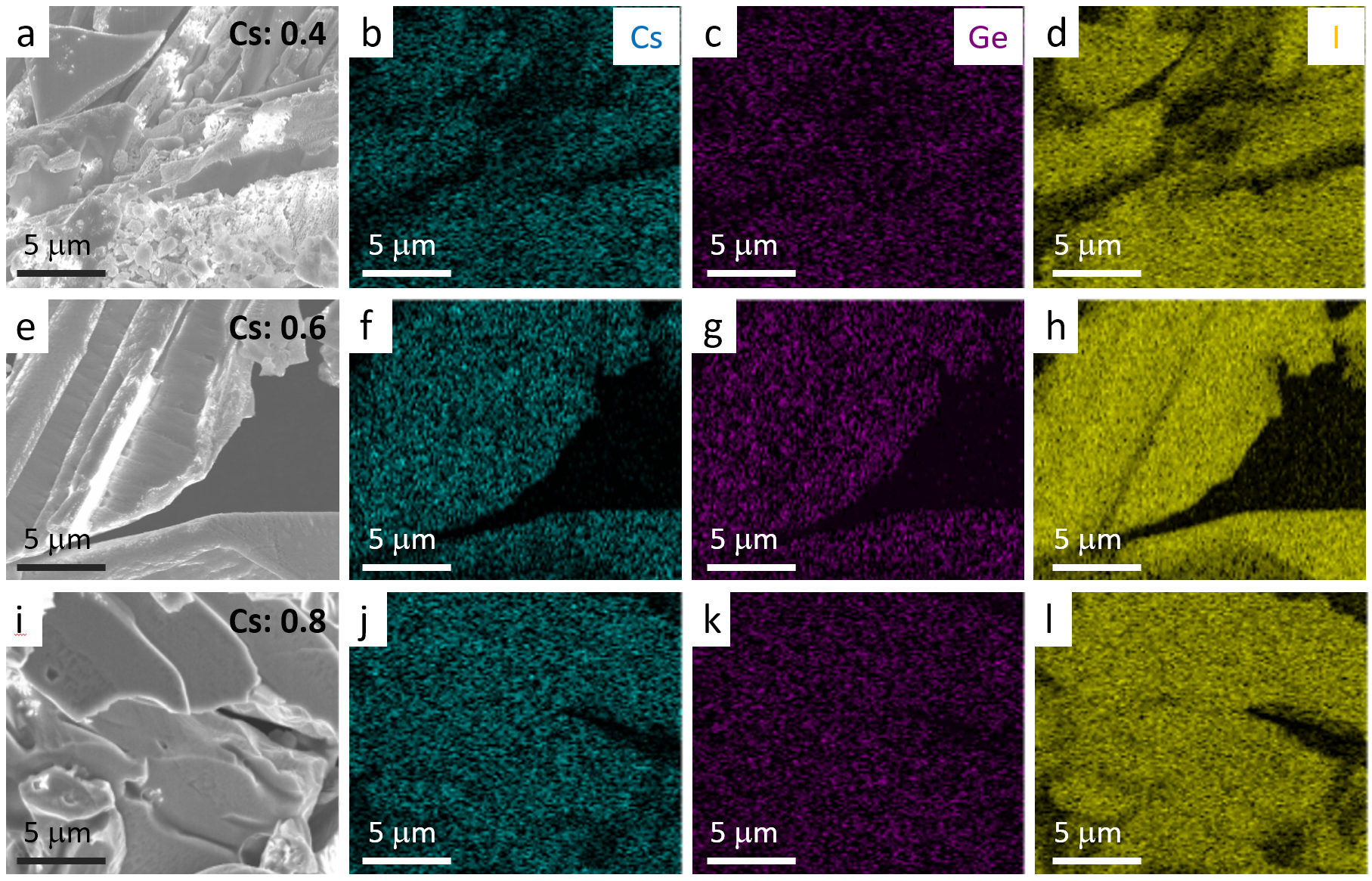


**Fig. S15 |** Scanning electron microscopy (SEM) images (a, e, i) of the Cs-substituted samples and the corresponding energy dispersive spectroscopy (EDS) mappings of Cs (b, f, j), Ge (c, g, k), and I (d, h, l) elements.


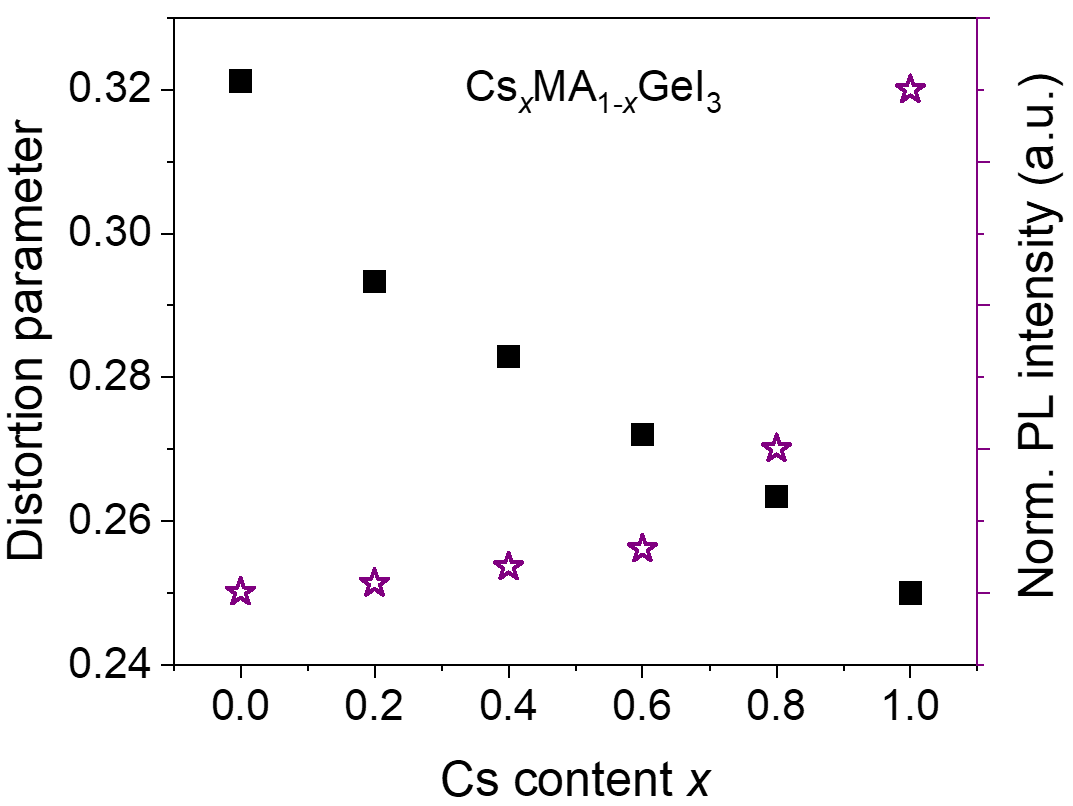


**Fig. S16 |** Distortion 𝒟 parameter and the spectrally integrated PL intensity of MA_1-_*_x_*Cs*_x_*GeI_3_ perovskites as a function of Cs concentration.


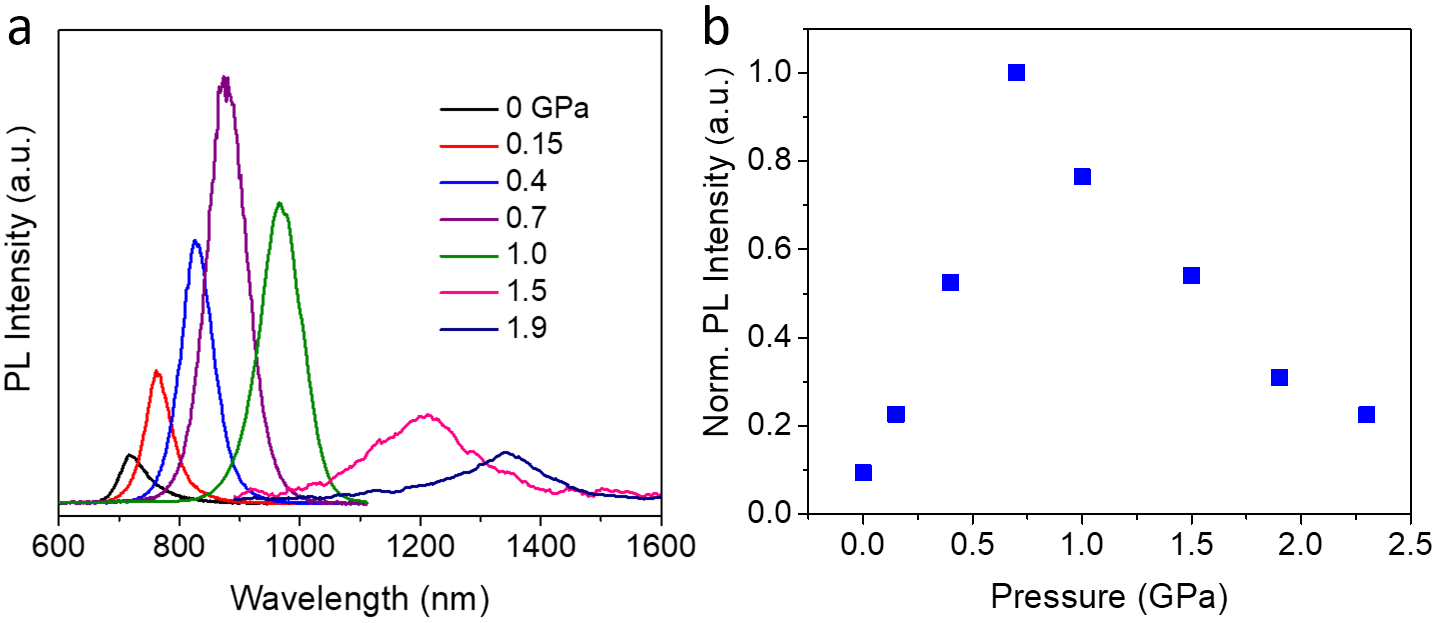


**Fig. S17 |** **Pressure-dependent photoluminescence properties of CsGeI_3_. a**, In situ PL spectra under high pressures. **b**, Pressure dependence of spectrally integrated PL emission intensity. The PL reaches the maximum value at 0.7 GPa and then weakens.


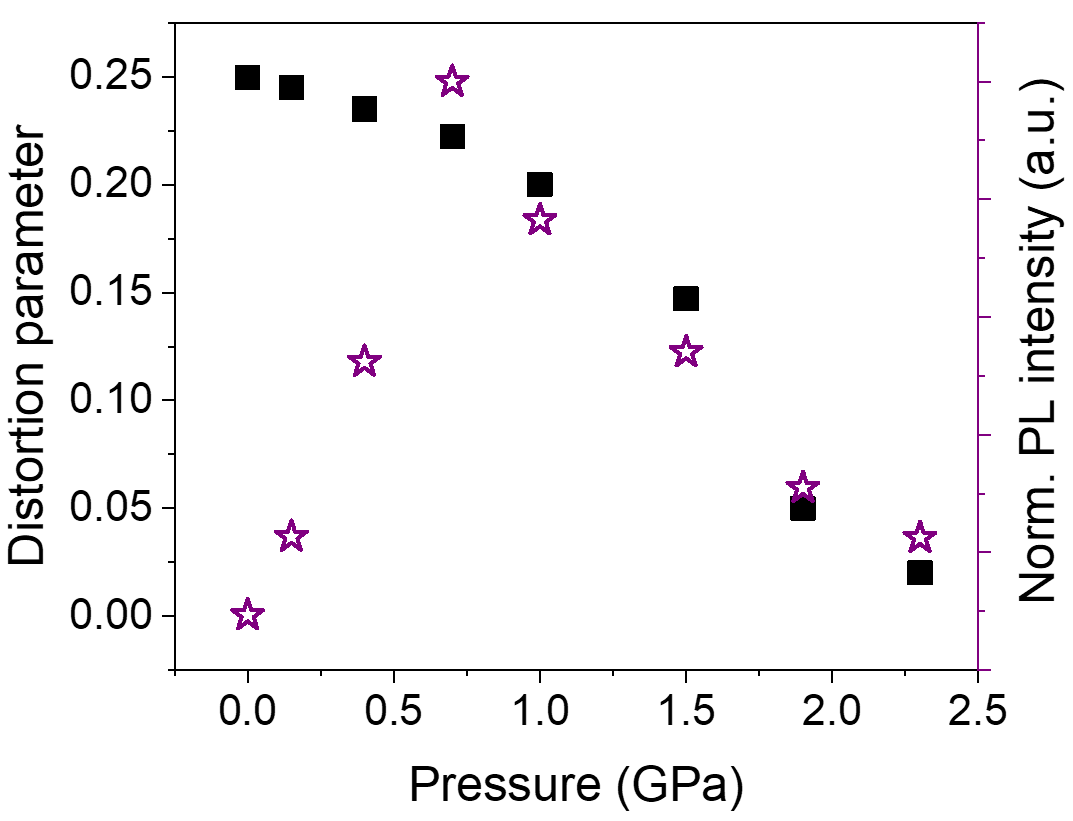


**Fig. S18 |** Distortion 𝒟 parameter of CsGeI_3_ under high pressures and the corresponding spectrally integrated PL intensity. 𝒟 value of CsGeI_3_ decreases with increasing pressure; while the emission strengthens first with pressure, reaches the maximum value at 0.7 GPa and then decreases.


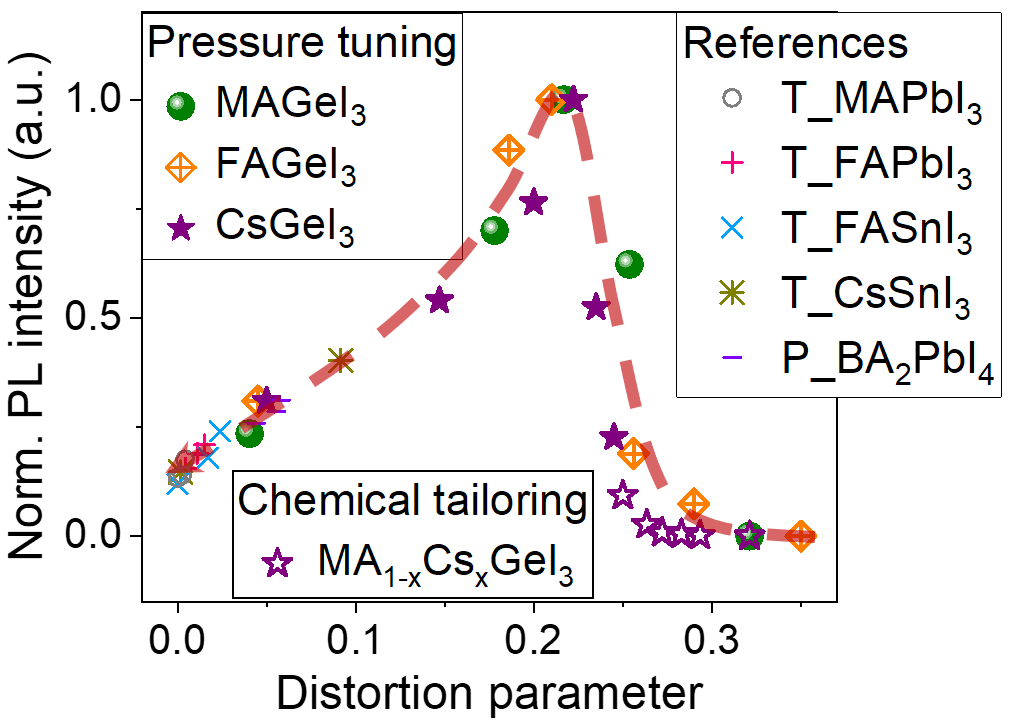


**Fig. S19.** **A universal relationship between structural distortion and PL intensity of halide perovskites.** It describes the pressure-tuned MAGeI_3_ (sphere), FAGeI_3_ (diamond), and CsGeI_3_ (solid star), as well as the chemical-tailored MA_1−x_Cs_x_GeI_3_ (open star). The cases regarding both temperature and pressure effects from the literature are also displayed. They include temperature effect on (CH_3_NH_3_)PbI_3_, [HC(NH_2_)_2_]PbI_3_, [HC(NH_2_)_2_]SnI_3_, and CsSnI_3_,[28, 38-42] and pressure effect on (C_4_H_9_NH_3_)_2_PbI_4_.[43] These systems possess high-quality crystallographic data and reliable PL intensity for determining the PL-𝒟 relationship, and they follow the principle revealed in this work nicely. Note that the distortion values for these previously reported systems are much smaller than those of the Ge halide perovskites. Regulating the octahedral distortion increases their PL intensity but has not reached the optimal distortion level demonstrated in the Ge halide perovskites in our study. Therefore, importantly, the highly distorted octahedra in Ge perovskites enable us to reach an otherwise unexplorable structural region, which provides a rare opportunity for understanding the structure-property relationship as well as optimizing the properties.

**REFERENCES**

1. Stoumpos, C. C.; Frazer, L.; Clark, D. J.; Kim, Y. S.; Rhim, S. H.; Freeman, A. J.; Ketterson, J. B.; Jang, J. I.; Kanatzidis, M. G., Hybrid Germanium Iodide Perovskite Semiconductors: Active Lone Pairs, Structural Distortions, Direct and Indirect Energy Gaps, and Strong Nonlinear Optical Properties. *J. Am. Chem. Soc.* 2015, **137**: 6804-6819.

2. Zhang, D.; Dera, P. K.; Eng, P. J.; Stubbs, J. E.; Zhang, J. S.; Prakapenka, V. B.; Rivers, M. L. High Pressure Single Crystal Diffraction at PX^2 *J. Vis. Exp.* [Online], 2017. PubMed. (accessed 2017/01//).

3. Dera, P.; Zhuravlev, K.; Prakapenka, V.; Rivers, M. L.; Finkelstein, G. J.; Grubor-Urosevic, O.; Tschauner, O.; Clark, S. M.; Downs, R. T., High pressure single-crystal micro X-ray diffraction analysis with GSE_ADA/RSV software. *High Pressure Res.* 2013, **33**: 466-484.

4. Sheldrick, G. M., A short history of SHELX. *Acta Crystallogr A.* 2008, **64**: 112-122.

5. Dolomanov, O. V.; Bourhis, L. J.; Gildea, R. J.; Howard, J. A.; Puschmann, H., OLEX2: a complete structure solution, refinement and analysis program. *J. Appl. Crystallogr.* 2009, **42**: 339-341.

6. Momma, K.; Izumi, F., VESTA: a three-dimensional visualization system for electronic and structural analysis. *J. Appl. Crystallogr.* 2008, **41**: 653-658.

7. Larson, A. C.; Von Dreele, R. B., General Structure Analysis System (GSAS), Los Alamos National Laboratory, Report LAUR, 86–748, 2000. *Los Alamos National Laboratory, Report LAUR* 2000: 86–748.

8. Tian, Y.; Merdasa, A.; Unger, E.; Abdellah, M.; Zheng, K.; McKibbin, S.; Mikkelsen, A.; Pullerits, T.; Yartsev, A.; Sundström, V.; Scheblykin, I. G., Enhanced Organo-Metal Halide Perovskite Photoluminescence from Nanosized Defect-Free Crystallites and Emitting Sites. *J. Phys. Chem. Lett.* 2015, **6**: 4171-4177.

9. Tian, Y.; Merdasa, A.; Peter, M.; Abdellah, M.; Zheng, K.; Ponseca, C. S.; Pullerits, T.; Yartsev, A.; Sundström, V.; Scheblykin, I. G., Giant Photoluminescence Blinking of Perovskite Nanocrystals Reveals Single-Trap Control of Luminescence. *Nano Lett.* 2015, **15**: 1603-1608.

10. Kresse, G.; Hafner, J., Ab initio molecular-dynamics simulation of the liquid-metal amorphous-semiconductor transition in germanium. *Phys. Rev. B* 1994, **49**: 14251-14269.

11. Perdew, J. P.; Burke, K.; Ernzerhof, M., Generalized Gradient Approximation Made Simple. *Phys. Rev. Lett.* 1996, **77**: 3865-3868.

12. Perdew, J. P.; Ruzsinszky, A.; Csonka, G. I.; Vydrov, O. A.; Scuseria, G. E.; Constantin, L. A.; Zhou, X.; Burke, K., Restoring the Density-Gradient Expansion for Exchange in Solids and Surfaces. *Phys. Rev. Lett.* 2008, **100**: 136406.

13. Steiner, S.; Khmelevskyi, S.; Marsmann, M.; Kresse, G., Calculation of the magnetic anisotropy with projected-augmented-wave methodology and the case study of disordered Fe_1-x_Co_x_ alloys. *Phys. Rev. B* 2016, **93**: 224425.

14. Setyawan, W.; Curtarolo, S., High-throughput electronic band structure calculations: Challenges and tools. *Comput. Mater. Sci.* 2010, **49**: 299-312.

15. Schouwink, P.; Ley, M. B.; Tissot, A.; Hagemann, H.; Jensen, T. R.; Smrčok, Ľ.; Černý, R., Structure and properties of complex hydride perovskite materials. *Nat. Commun.* 2014, **5**: 5706.

16. Leblebici, S. Y.; Leppert, L.; Li, Y.; Reyes-Lillo, S. E.; Wickenburg, S.; Wong, E.; Lee, J.; Melli, M.; Ziegler, D.; Angell, D. K.; Ogletree, D. F.; Ashby, Paul D.; Toma, F. M.; Neaton, J. B.; Sharp, I. D.; Weber-Bargioni, A., Facet-dependent photovoltaic efficiency variations in single grains of hybrid halide perovskite. *Nat. Energy* 2016, **1**: 16093.

17. Draguta, S.; Sharia, O.; Yoon, S. J.; Brennan, M. C.; Morozov, Y. V.; Manser, J. S.; Kamat, P. V.; Schneider, W. F.; Kuno, M., Rationalizing the light-induced phase separation of mixed halide organic–inorganic perovskites. *Nat. Commun.* 2017, **8**: 200.

18. Meng, W.; Wang, X.; Xiao, Z.; Wang, J.; Mitzi, D. B.; Yan, Y., Parity-Forbidden Transitions and Their Impact on the Optical Absorption Properties of Lead-Free Metal Halide Perovskites and Double Perovskites. *J. Phys. Chem. Lett.* 2017, **8**: 2999-3007.

19. Wu, B.; Yuan, H.; Xu, Q.; Steele, J. A.; Giovanni, D.; Puech, P.; Fu, J.; Ng, Y. F.; Jamaludin, N. F.; Solanki, A.; Mhaisalkar, S.; Mathews, N.; Roeffaers, M. B. J.; Grätzel, M.; Hofkens, J.; Sum, T. C., Indirect tail states formation by thermal-induced polar fluctuations in halide perovskites. *Nat. Commun.* 2019, **10**: 484.

20. Garten, L. M.; Moore, D. T.; Nanayakkara, S. U.; Dwaraknath, S.; Schulz, P.; Wands, J.; Rockett, A.; Newell, B.; Persson, K. A.; Trolier-McKinstry, S.; Ginley, D. S., The existence and impact of persistent ferroelectric domains in MAPbI_3_. *Sci. Adv.* 2019, **5**: eaas9311.

21. Jiang, J.; Sun, X.; Chen, X.; Wang, B.; Chen, Z.; Hu, Y.; Guo, Y.; Zhang, L.; Ma, Y.; Gao, L.; Zheng, F.; Jin, L.; Chen, M.; Ma, Z.; Zhou, Y.; Padture, N. P.; Beach, K.; Terrones, H.; Shi, Y.; Gall, D.; Lu, T.-M.; Wertz, E.; Feng, J.; Shi, J., Carrier lifetime enhancement in halide perovskite via remote epitaxy. *Nat. Commun.* 2019, **10**: 4145.

22. Feldmann, S.; Macpherson, S.; Senanayak, S. P.; Abdi-Jalebi, M.; Rivett, J. P. H.; Nan, G.; Tainter, G. D.; Doherty, T. A. S.; Frohna, K.; Ringe, E.; Friend, R. H.; Sirringhaus, H.; Saliba, M.; Beljonne, D.; Stranks, S. D.; Deschler, F., Photodoping through local charge carrier accumulation in alloyed hybrid perovskites for highly efficient luminescence. *Nat. Photonics* 2020, **14**: 123-128.

23. Zimmermann, N. E. R.; Hannah, D. C.; Rong, Z.; Liu, M.; Ceder, G.; Haranczyk, M.; Persson, K. A., Electrostatic estimation of intercalant jump-diffusion barriers using finite-size ion models. *J. Phys. Chem. Lett.* 2018, **9**: 628-634.

24. Bechtel, J. S.; Van der Ven, A., Octahedral tilting instabilities in inorganic halide perovskites. *Phys. Rev. Mater.* 2018, **2**: 025401.

25. Kim, J.; Lee, S.-H.; Lee, J. H.; Hong, K.-H., The Role of Intrinsic Defects in Methylammonium Lead Iodide Perovskite. *J. Phys. Chem. Lett.* 2014, **5**: 1312-1317.

26. Ledinský, M.; Löper, P.; Niesen, B.; Holovský, J.; Moon, S.-J.; Yum, J.-H.; De Wolf, S.; Fejfar, A.; Ballif, C., Raman Spectroscopy of Organic–Inorganic Halide Perovskites. *J. Phys. Chem. Lett.* 2015, **6**: 401-406.

27. Yaffe, O.; Guo, Y.; Tan, L. Z.; Egger, D. A.; Hull, T.; Stoumpos, C. C.; Zheng, F.; Heinz, T. F.; Kronik, L.; Kanatzidis, M. G.; Owen, J. S.; Rappe, A. M.; Pimenta, M. A.; Brus, L. E., Local Polar Fluctuations in Lead Halide Perovskite Crystals. *Phys. Rev. Lett.* 2017, **118**: 136001.

28. Stoumpos, C. C.; Malliakas, C. D.; Kanatzidis, M. G., Semiconducting Tin and Lead Iodide Perovskites with Organic Cations: Phase Transitions, High Mobilities, and Near-Infrared Photoluminescent Properties. *Inorg. Chem.* 2013, **52**: 9019-9038.

29. Green, M. A.; Ho-Baillie, A.; Snaith, H. J., The emergence of perovskite solar cells. *Nat. Photonics* 2014, **8**: 506-514.

30. Stranks, S. D.; Burlakov, V. M.; Leijtens, T.; Ball, J. M.; Goriely, A.; Snaith, H. J., Recombination Kinetics in Organic-Inorganic Perovskites: Excitons, Free Charge, and Subgap States. *Phys. Rev. Appl.* 2014, **2**: 034007.

31. Hoke, E. T.; Slotcavage, D. J.; Dohner, E. R.; Bowring, A. R.; Karunadasa, H. I.; McGehee, M. D., Reversible photo-induced trap formation in mixed-halide hybrid perovskites for photovoltaics. *Chem. Sci.* 2015, **6**: 613-617.

32. Ball, J. M.; Petrozza, A., Defects in perovskite-halides and their effects in solar cells. *Nat. Energy* 2016, **1**: 16149.

33. Oga, H.; Saeki, A.; Ogomi, Y.; Hayase, S.; Seki, S., Improved Understanding of the Electronic and Energetic Landscapes of Perovskite Solar Cells: High Local Charge Carrier Mobility, Reduced Recombination, and Extremely Shallow Traps. *J. Am. Chem. Soc.* 2014, **136**: 13818-13825.

34. Yin, W.-J.; Shi, T.; Yan, Y., Unusual defect physics in CH3NH3PbI3 perovskite solar cell absorber. *Appl. Phys. Lett.* 2014, **104**: 063903.

35. Lin, T.; Yang, C.; Wang, Z.; Yin, H.; Lu, X.; Huang, F.; Lin, J.; Xie, X.; Jiang, M., Effective nonmetal incorporation in black titania with enhanced solar energy utilization. *Energ. Environ. Sci.* 2014, **7**: 967-972.

36. De Wolf, S.; Holovsky, J.; Moon, S.-J.; Löper, P.; Niesen, B.; Ledinsky, M.; Haug, F.-J.; Yum, J.-H.; Ballif, C., Organometallic halide perovskites: sharp optical absorption edge and its relation to photovoltaic performance. *J. Phys. Chem. Lett.* 2014, **5**: 1035-1039.

37. Kong, L.; Liu, G.; Gong, J.; Hu, Q.; Schaller, R. D.; Dera, P.; Zhang, D.; Liu, Z.; Yang, W.; Zhu, K.; Tang, Y.; Wang, C.; Wei, S.-H.; Xu, T.; Mao, H.-k., Simultaneous band-gap narrowing and carrier-lifetime prolongation of organic–inorganic trihalide perovskites. *Proc. Natl. Acad. Sci. U.S.A.* 2016, **113**: 8910-8915.

38. Liu, Y.; Lu, H.; Niu, J.; Zhang, H.; Lou, S.; Gao, C.; Zhan, Y.; Zhang, X.; Jin, Q.; Zheng, L., Temperature-dependent photoluminescence spectra and decay dynamics of MAPbBr3 and MAPbI3 thin films. *AIP Adv.* 2018, **8**: 095108.

39. Weller, M. T.; Weber, O. J.; Henry, P. F.; Di Pumpo, A. M.; Hansen, T. C., Complete structure and cation orientation in the perovskite photovoltaic methylammonium lead iodide between 100 and 352 K. *Chem. Commun.* 2015, **51**: 4180-4183.

40. Francisco-López, A.; Charles, B.; Alonso, M. I.; Garriga, M.; Campoy-Quiles, M.; Weller, M. T.; Goñi, A. R., Phase Diagram of Methylammonium/Formamidinium Lead Iodide Perovskite Solid Solutions from Temperature-Dependent Photoluminescence and Raman Spectroscopies. *J. Phys. Chem. C* 2020, **124**: 3448-3458.

41. Schueller, E. C.; Laurita, G.; Fabini, D. H.; Stoumpos, C. C.; Kanatzidis, M. G.; Seshadri, R., Crystal Structure Evolution and Notable Thermal Expansion in Hybrid Perovskites Formamidinium Tin Iodide and Formamidinium Lead Bromide. *Inorg. Chem.* 2018, **57**: 695-701.

42. Kontos, A. G.; Kaltzoglou, A.; Arfanis, M. K.; McCall, K. M.; Stoumpos, C. C.; Wessels, B. W.; Falaras, P.; Kanatzidis, M. G., Dynamic Disorder, Band Gap Widening, and Persistent Near-IR Photoluminescence up to At Least 523 K in ASnI3 Perovskites (A = Cs^+^, CH_3_NH_3_^+^ and NH_2_–CH═NH_2_^+^). *J. Phys. Chem. C* 2018, **122**: 26353-26361.

43. Yin, T.; Liu, B.; Yan, J.; Fang, Y.; Chen, M.; Chong, W. K.; Jiang, S.; Kuo, J.-L.; Fang, J.; Liang, P.; Wei, S.; Loh, K. P.; Sum, T. C.; White, T. J.; Shen, Z. X., Pressure-Engineered Structural and Optical Properties of Two-Dimensional (C_4_H_9_NH_3_)_2_PbI_4_ Perovskite Exfoliated nm-Thin Flakes. *J. Am. Chem. Soc.* 2019, **141**: 1235-1241.
